# Working capital management, financial constraints and exports: evidence from European and US manufacturers

**DOI:** 10.1007/s00181-022-02295-5

**Published:** 2022-08-23

**Authors:** José Manuel Mansilla-Fernández, Juliette Milgram-Baleix

**Affiliations:** 1 Department of Business Management and Institute for Advanced Research in Business and Economics (INARBE), Public University of Navarre (UPNA), Arrosadia Campus, 31006 Pamplona, Navarre Spain; 2 Department of Economics Theory and Economic History, University of Granada, Campus Universitario de Cartuja, 10587 Granada, Granada Spain

**Keywords:** Cash conversion cycle, COVID-19 crisis, Exports, Financial constraints, Working capital management, G01, G21, G32, H63

## Abstract

This paper investigates the effect of firms’ working capital management, measured by the cash conversion cycle (CCC) on exports, on both the intensive and extensive margins. By using Heckman’s two-stage model for the treatment of sample selection bias, we find that the longer the CCC, the lower firms’ likelihood of exporting and the lower the volume of their exports. This phenomenon is economically more relevant for financially constrained firms than for unconstrained firms. The results are robust to the propensity score matching, the transition sample and the placebo analyses. Finally, these results can be extrapolated in the context of the COVID-19 crisis because of the decline in trading conditions and firms’ shortage of liquidity.

## Introduction

In the aftermath of the global financial crisis, international trade collapsed drastically worldwide both in advanced and in emerging economies. The downturn in international trade was substantially larger than the drop in manufacturing production, raising the natural question of the role played by trade finance in the ‘Great Trade Collapse’ (see Ahn et al. 2011; Alessandria et al. 2010; Amiti and Weinstein 2011; Bricongne et al. 2012; Chor and Manova 2012; Manova 2013; Antràs and Foley 2015). Understanding to what extent the fall in credit availability due to the banking crisis has been responsible for the drop in trade has important policy implications since it would indicate that recovery is linked to the return of trade credit (see Alessandria et al. 2010). A more worrisome consequence would be that a similar disproportionate trade response can be expected following financial perturbations. In the recent context of COVID-19 pandemic, the great lockdown and further containment measures are likely to shatter the productive sector and households’ behaviour (Atkeson 2020; Bodenstein et al. 2020). The latest forecasts on production and (un)employment point out a possible increase in impairment loans and public debt (see Angelini et al. 2020), which might harm banks’ balance sheets. As a result, banks might face rising financing costs that would reduce credit availability to the real sector (Chiesa and Mansilla-Fernández 2019, 2021; Li et al. 2020). In this scenario, further firms might find difficulties accessing bank credit and become financially constrained.^1^ Importantly, poor trading conditions—e.g. a drop in sales or disruptions in payment chains—put pressure on firms’ liquidity, which reflects their capacity to manage working capital to export and avoid bankruptcies on a large scale (e.g. Ferrando and Ganoulis 2020; Schivardi and Romano 2020).

In the last decade, the theoretical and empirical literature has proposed and corroborated several explanations for the puzzling fact that banking crises have a more substantial effect on international trade than on domestic activities (e.g. Foley and Manova 2015, for a survey). The main explanation comes from the fact that selling abroad implies additional costs compared to domestic sales. Firms entering foreign markets have to engage in a series of activities that are related only to exporting, such as market research, setting up a new distribution network, learning about administrative standards, negotiating with potential new partners and modifying existing product ranges (see Albornoz et al. 2012). These costs are mainly sunk in nature and affect entry into the export market (see Roberts and Tybout 1997). This is also the main reason why the most productive firms self-select into exporting (Melitz 2003) and why firm heterogeneity has been proven to be a key determinant of aggregate exports. Apart from these sunk costs, exporters face other additional costs related to the fact that international shipments take more time and cross-border relationships are riskier. The delay between the payment and the delivery of goods is longer, contract enforcement is less guaranteed, and exporters face the additional risk of damage and additional insurance costs (Doan et al. 2020). Financing trade transactions thus faces more obstacles than financing domestic ones. As a matter of fact, introducing the need for external finance to cope with these additional fixed and variable costs leads theoretical models to predict that less financially constrained firms are more likely to export (Chaney 2016) and would export larger amounts (Manova 2013) than financially constrained firms. On the empirical side, starting with Greenaway et al. (2007), many studies have focused on the relationship between financial constraints and export behaviour using firm-level data and tend to corroborate the theoretical predictions (see, e.g. Pietrovito and Pozzolo 2021; Wagner 2014; Foley and Manova 2015, for a survey). However, most studies focus only on one country, except (Doan et al. 2020), thus reducing the possibility of generalizing their results.

To the best of our knowledge, little attention has been paid to the role of working capital in funding exports, even though the aforementioned literature indirectly suggests that working capital—or liquidity—management could be crucial for exporters, both on the intensive and on the extensive margins. Surprisingly, most studies have focused on external financing while the role played by working capital management has been overlooked in the literature. The only articles tackling this issue we are aware of are Ahn et al. (2011) and Alessandria et al. (2010), who show that inventory dynamics play an important role in explaining the volatility of trade, and Doan et al. (2020), who study the role of cash in advance, a form of trade credit on the participation of small- and medium-sized firms in exports. If exporters rely on inventories in response to supply or demand shocks, the most common source of internal financing for exporters comes from credits between exporters and importers. Exporters have to reduce the length of time between the moment they pay their inputs that may be imported and the moment they obtain payment for their sales. Unlike previous studies, this article focuses on the capacity of generating internal funds through working capital management—that is, reducing inventories and limiting the amount of trade credits to their clients while extending trade debit to their providers. Actually, granting [lengthy] trade credit or stocking inventories generates opportunity costs for exporters, which would divert investments away from internationalization (Deloof 2003). Likewise, delaying payments to providers fosters firms’ exports, since payables are a relatively cheap source of financing (Long et al. 1993). Furthermore, firms receiving trade finance are less likely to cause moral hazard and banks would be more willing to grant them credit (Doan et al. 2020). In line with studies advocating the Pecking Order theory (Myers 1984; Myers and Majluf 1984) and previous literature linking financial restrictions to export behaviour, we argue that the ability of firms to finance short-term debt internally may play an important role in their capacity to export. Additionally, we conjecture that the contribution of liquidity to exports may be even more relevant for firms facing financial constraints.

To study this question, we use a large sample of European and US manufacturing firms for the period 2012–2020 retrieved from *Bureau van Dijk*’s Orbis database. We measure working capital management using the cash conversion cycle (CCC hereafter) which represents firms’ need for short-term debt financing. CCC is computed as the number of days that trade credit and inventories outstanding convert into cash, minus the days that payables outstanding do (e.g. Zeidan and Shapir 2017; Wang 2019). Accordingly, the shorter the CCC, the better the management of working capital. Financial constraints and length of working capital may both affect the financing of fixed costs but also variable costs, thus affecting both the decision to enter a market (extensive margin) and the size of foreign shipments (intensive margin). First, we test whether a shorter CCC fosters entry into foreign markets and increase firms’ exports. We quantify these two mechanisms with a two-stage estimation procedure (Heckman 1979) to control for the fact that firms self-select into exports. Second, the transmission channel is disentangled by looking into the components of the CCC. Third, we investigate whether working capital management is economically more important for constrained than for unconstrained exporters. For this purpose, we rely on three alternative indicators for financial constraints: (i) the Hadlock and Pierce (2010) SA index, (ii) the Whited and Wu (2006) index and (iii) the interest burden index (e.g. Fernandes et al. 2019; Guariglia et al. 2016; Mulier et al. 2016). To preview our results, our main hypothesis is confirmed. We do indeed find that firms performing better in terms of working capital management (that is, with shorter CCC) enjoy a higher probability of exports and sell larger amounts compared to other exporters. As expected, trade credit and inventory periods reduce the probability of exporting and the volume of exports, while delaying payments to providers fosters firms’ exports.

In line with the previous research, our results confirm the idea that financial constraints impact negatively on exports. We add to the literature by showing that the impact of the CCC is comparatively higher for financially constrained firms, which means that the lack of internal finance makes firms more dependent on liquidity to export.

Finally, we carry out a propensity score matching analysis to check for observed differences between financially constrained and unconstrained firms. Importantly, we investigate a sample of transitioning firms that change their status from unconstrained to constrained. This test is aimed at controlling for the effect of time-invariant unobservable firm characteristics. In particular, we find that export revenues start to struggle when firms experience their first constraint event, but this impact vanishes over time. Lastly, we run a placebo test to check for possible misidentification issues. Overall, the estimates for CCC and for the control variables are consistent with the baseline results.

The rest of the article proceeds as follows: Section 2 discusses the theoretical background of this study; Section 3 presents the hypotheses, the data and the empirical framework of this research; Section 4 describes the main results; and Section 5 concludes.

## Literature review

This research builds on the literature linking finance frictions with trade activities on the one hand and on the literature linking firms’ financial needs with working capital management on the other. An important seminal contribution is the theoretical model of Kletzer and Bardhan (1987), who investigate credit markets’ imperfections when credit for working capital and trade finance are needed. Importantly, the Kletzer and Bardhan model shows that credit market imperfections translate into differences in interest rates and credit constraints between countries that generate, per se, differences in costs between countries, all else being equal. Before the global financial crisis, Chaney (2005) showed that liquidity constraints affect entry into the export market in trade models with heterogeneity of firms, and Greenaway et al. (2007) were pioneers in testing empirically the link between firms’ financial health and their export behaviour. Following the ‘Great Trade Collapse’, the literature has put greater emphasis on studying why financial problems would hurt international transactions more heavily than domestic ones.

Overall, the literature demonstrates that exporting activities are highly dependent on external financial resources. From a theoretical perspective, the need to finance fixed and variable costs associated with exporting activities provides a potential explanation for why international trade is significantly affected by external financial conditions. As suggested by Roberts and Tybout (1997), to enter foreign markets exporters face irreversible investments related to market research, adaptation to technical and administrative standards, the search for distribution networks, negotiations with potential new partners, the modification of existing product ranges, etc. Only the most productive firms are able to cope with these sunk costs and enter the export markets (Melitz 2003). Apart from these upfront costs, exporters also face greater variable costs, such as transportation costs, duties and freight insurance. They also face additional risks due to currency fluctuations and difficulties in enforcing contracts that involve different jurisdictions, as a foreign partner is particularly difficult to monitor (see Ellingsen and Vlachos 2011). Exporters also have higher working capital needs relative to those of domestic manufacturers due to longer shipping times (see Doan et al. 2020; Foley and Manova 2015).

To afford these extra costs, firms require external financing, which leads one to predict that only non-financially constrained firms can access foreign markets and that they export larger amounts than financially constrained ones (see Muûls 2015; Chaney 2016; Manova 2013). Based on the above-mentioned models, empirical studies using firm-level data have investigated the channel through which shortage of credit or lack of liquidity diminish international trade. See, e.g. Wagner 2014; Foley and Manova 2015; Pietrovito and Pozzolo 2021, for a survey. With a few exceptions, these studies converge on the conclusion that inaccessibility to external finance contributes to reducing firms’ likelihood of becoming exporters and the volume of foreign sales.^2^ Most of these studies focus on specific countries, such as that done recently by Máñez and Vicente-Chirivella (2020) and Mukherjee and Chanda (2021), while very few studies have used firm-level datasets involving several developing or emerging countries, such as Berman and Héricourt (2010), Jinjarak and Wignaraja (2016) and Pietrovito and Pozzolo (2021).

Importantly, Pietrovito and Pozzolo (2021) confirm previous results and provide a methodological contribution to address potential endogeneity issues derived from the effects of credit constraints on exports. These authors add to this literature by showing the dynamic effects of financial constraints on the volume of exports. Unlike Besedeš et al. (2014), who use years exporting as a duration variable, this paper investigates the dynamic impact of financial constraints (year-by-year since the first event) on the volume of export sales.

The above articles focus mainly on the possibility firms have of accessing external credits but do not consider the role played by trade-specific financial instruments. However, exporters usually resort to trade credits, either through cash in advance or under open-account terms,^3^ or to intermediates to obtain letters of credit.^4^

We intend to fill this gap by focusing on the role played by working capital management as capacity to generate internal funds in firms’ export behaviour. Working capital management consists of the management of inventories, accounts receivable and accounts payable to cover the payment of inputs before obtaining output revenues. This question has been completely overlooked in the literature except by Doan et al. (2020), who study the role of cash in advance on the participation of small- and medium-sized firms in exports from 56 developing countries using data from the World Bank Enterprise Survey. We base our study on the Pecking Order theory of capital structure, which establishes that firms prefer internal to external capital resources due to adverse selection (Myers 1984; Myers and Majluf 1984). Indeed, if firms delay their collection periods to their customers or accumulate large stocks, they might increase their sales in the short term, but assuming the opportunity costs of taking impairment risk and postponing investments in fixed capital (Deloof 2003). On the other hand, delaying payments to suppliers can be an inexpensive and flexible financial source for the firm (Long et al. 1993).^5^

The growing literature on working capital management tends to conclude that the CCC is a powerful predictor of firms’ financing needs (Deloof 2003; Zeidan and Shapir 2017). The CCC is the kernel of the transmission channel under investigation for two reasons. First, for technological reasons, the length of the production processes determines firms’ capacity to stock and sell, thus lengthening the CCC. Second, the higher the CCC, the higher a firm’s necessity to finance working capital and to rely on short-term debt (Wang 2019). If funding liquidity deterioration complicates fund-raising or causes losses in rolling over debt maturity, firms can be relatively over-exposed to aggregate risk. Interestingly, recent research demonstrates that comparatively high-CCC firms are more dependent on external financing, which increases their vulnerability (Raddatz 2006; Tong and Wei 2011). Therefore, this article endeavours to contribute to the existent literature by showing that firms’ capacity to export is determined by the length of their CCC. To the best of our knowledge, this is one of the first articles investigating the relationship between working capital management—and particularly the CCC—and exports. Thus, the first hypothesis of this study can be formulated as follows:

### Hypothesis 1

The higher the cash conversion cycle (CCC), the lower a firm’s probability of exporting and the lower a firm exports.

The second research question is whether financial constraints might accentuate the impact of the CCC on exports. This article is aligned with previous research demonstrating the importance of liquidity, particularly when capital markets are imperfect (Blanchard et al. 1994; Kim et al. 1998; Lins et al. 2010; Yun 2009).^6^ Along the same lines, Almeida et al. (2004, 2011), employing several financial constraint criteria, demonstrate that financially constrained firms tend to save more cash than non-financially constrained ones. The level of sensitivity is affected by the future level of investment opportunities captured partly by cash flow (Machokoto and Areneke 2020). On the other hand, firms with high credit rating are found to access financial markets more easily and thus need to hold lower levels of cash flow than constrained ones (Mulier et al. 2016). Denis and Sibilkov (2009) support those results and demonstrate that greater cash holdings are associated with higher levels of investment for constrained firms, whereas that value is also stronger than for unconstrained ones. Likewise, Bigelli and Sánchez-Vidal (2012) show that more cash is also held by firms with longer cash conversion cycles and lower financing deficits. Constrained firms also burned through cash and drew more heavily on lines of credit for fear of banks. Besides, they would have to sell more assets to fund their operations (Campello et al. 2010). Interestingly, trade creditors are usually capable of monitoring the financial health of their customers, before deciding to finance them (e.g. Burkart and Ellingsen 2004; Aktas et al. 2012; Yang 2011). Put in other words, the sensitivity of exports to working capital is expected to intensify with credit constraints. Thus, the second hypothesis of this study can be stated as follows:

### Hypothesis 2

Financial constraint heightens the impact of CCC on firms’ exports.

To sum up, this investigation aims to contribute to the existing literature by showing that working capital management is a determinant of exports. Importantly, this article hypothesizes that financial constraints might catalyse the effect of CCC on exports and on the probability of firms’ internationalization, i.e. working capital management is hypothesized to be economically more relevant for constrained than for unconstrained firms.

## Data and methodology

This section describes the construction of the dataset, the variables and the baseline model to test the hypotheses of this research.

### Data and sample selection

The main data source for firm-level information is *Bureau van Dijk’s* Orbis database. This corporate database contains comparable financial and managerial information for companies worldwide and has been used extensively in studies dealing with multinational companies (Fariñas et al. 2018; Weche 2018).^7^

The sample consists of consolidated accounting data on European and US manufacturers for the period 2012–2020. All the companies included in the dataset provide data from 1st January to 31st December. Since Orbis provides information on export revenues, we are able to distinguish between exporters and non-exporters. To ensure that the identification is accurate, we drop companies with unknown values of export revenues from the sample. Companies are selected at the highest possible level of consolidation, usually as holding groups, to avoid double-entry issues. Firms that do not belong to any holding groups are considered single companies.

We consider the years 2012–2020 as appropriate to our purpose since they include the period immediately after the financial crisis, which resulted in a shock in credit markets that contributed to diminishing multinational activities (e.g. Manova et al. 2015).

The data are expressed in euros and deflated by the Harmonized Consumer Price Index (HCPI). Inconsistent observations such as zero for either total assets, capital or workers are removed from the sample. The final dataset consists of a sample of 13,727 manufacturing companies from Croatia, Estonia, France, Germany, Hungary, Ireland, the UK and the USA. This yields a balanced panel of 123,543 observations. Macroeconomic information is retrieved from Eurostat at the regional (NUTS1) level. Macroeconomic variables are merged with the Orbis database, which also provides this information.

**Table 1:** Variable definitions

Variable	Acronym	Definition
*Dependent variables*		
Volume of exports	$\textrm{Ln}(E_{it})$	Natural logarithm of export sales ($E_{it}$)
Export status	$ExpD_{it}$	This dummy variable takes the value one if firm *i* exports in year *t*, and zero, otherwise
*Working capital management indicators*		
Trade credit period	$\textrm{TC}_{it}$	This variable measures the number of days that firm grants trade credit to customers. This indicator is measured as *account receivables* over *sales* multiplied by 365 days
Inventory period	$\textrm{INV}_{it}$	This variable measures the number of days that the firm accumulates stocks. Inventories are measured directly from the balance sheet. This indicator is constructed as the *inventories* to *cost of goods sold* ratio multiplied by 365
Trade debit period	$\textrm{TD}_{it}$	This variable measures the number of days that firm requires for collecting trade credit and indicates the effectiveness with which a firm manages granting credit. This indicator is measured as the *account payables* to *cost of goods sold* ratio multiplied by 365 days
Cash Conversion Cycle	$\textrm{CCC}_{it}$	This indicator measures the quality of working capital management. The cash conversion cycle is computed as the sum of above-mentioned tree indicators ($\textrm{CCC}_{it} = \textrm{TC}_{it} + \textrm{INV}_{it} - \textrm{TD}_{it}$)
*Control variables*		
Firm’s experience	$\textrm{Age}_{i,t-1}$	This indicator is constructed as the natural logarithm of the difference between the current (*t*) and the founding year
Employees	$\textrm{Ln}(\textrm{Emp}_{it})$	This indicator is constructed as the natural logarithm of the number of employees
Business generation	$\textrm{CFK}_{it}$	This variable is measured as the cash-flow-to-fixed-assets ratio
Leverage	$\textrm{LEV}_{it}$	This indicator is constructed as the liabilities to equity ratio plus one
Total Factor Productivity	$\textrm{Ln}(\textrm{TFP}_{it})$	This indicators controls for firm’s productivity. TFP is computed following the Levinsohn and Petrin (2003) methodology
Covid	$\textrm{Covid}_{t}$	This dummy variable controls for the outbreak of the COVID-19 pandemic and takes the value one if $t=2020$, and zero, otherwise
GDP growth	$\textrm{GDP}_{ht}$	This variable controls for the regional economic cycle and it is measured as the growth rate of the GDP at the NUTS 2 level (*h*)
*Financial constraint indicators*		
SA index	$\textrm{SAD}_{it}$	The Hadlock and Pierce (2010) SA index is constructed as
		$$\textrm{SA}_{it}=-0.737\textrm{Ln}(\textrm{TA}_{it}) + 0.043\left( \textrm{Ln}(\textrm{TA}_{it})\right) ^2- 0.040\textrm{Age}_{it}$$
		Where $\textrm{Ln}(\textrm{TA}_{it})$ is the natural logarithm of firm’s total assets ($\textrm{TA}_{it}$)
		The indicator $\textrm{SAD}_{it} = 1$ if percentile $> 1/3$, and zero otherwise
WW index	$\textrm{WWD}_{it}$	The Whited and Wu (2006) (WW) index is computed as $\textrm{WW}_{it} -0.091\textrm{CFTA}_{it}-0.062\textrm{DIVPOS}_{it}+0.021\textrm{TLTD}_{it}-0.044\textrm{Ln}(\textrm{TA}_{it}) + 0.102\textrm{ISG}_{kt} - 0.035\textrm{SG}_{it}$
		The definition of the factors proceed as follow:
		$$\textrm{CFTA}_{it} = \text { Cash-flow}_{it} / \textrm{TA}_{it}$$
		$\textrm{DIVPOS}_{it} = 1$ if *retained earnings* in year $t+1$ exceeds net income in year *t*
		$$\textrm{TLTD}_{it} = \text { Long-term debt}_{it} / \textrm{TA}_{it}$$
		$\textrm{ISG}_{kt}$ is the variation rate of added value at the industrial level
		$\textrm{SG}_{it}$ is the variation rate of firm’s sales
		The indicator $\textrm{WWD}_{it}=1$ if percentile $> 1/3$, and zero otherwise
IB index	$\textrm{IBD}_{it}$	The interest burden ratio ($\textrm{IB}_{it}$) is defined as the *interest paid* to *cash-flow* ratio
		The indicator $\textrm{IBD}_{it}=1$ if percentile $> 1/3$, and zero otherwise

*i*, *h* and *t* subscripts refer to firm, region and time, respectively

Finally, Table 1 contains the definitions and explanations of all the variables used in this article, all of which are winsorized at 1%.

### The cash conversion cycle

This article uses the cash conversion cycle as a measure of working capital management. This indicator represents the length of time taken by the company to sell inventories and collect receivables compared with the length of time during which the firm pays its trade debit. In other words, the CCC captures the length of period in which firm liquidity is bound to the business. This indicator has been widely used to assess firms’ dependence on external financing for working capital (e.g. Baños-Caballero et al. 2012; Wang 2019; Zeidan and Shapir 2017). The cash conversion cycle ($\textrm{CCC}_{it}$) is calculated as follows:

1 $$\begin{aligned} \begin{aligned} \textrm{CCC}_{it}&= 365\times \left( \frac{\textrm{AR}_{it}}{\textrm{Sales}_{it}} + \frac{I_{it}}{\textrm{COGS}_{it}} - \frac{\textrm{AP}_{it}}{\textrm{COGS}_{it}} \right) \\&= \textrm{TC}_{it} + \textrm{INV}_{it} - \textrm{TD}_{it} \end{aligned} \end{aligned}$$

where the subscripts *i* and *t* denote the firm and the year, respectively. The variable $\textrm{CCC}_{it}$ comprises all the steps of the production process. First, the trade credit period ($\textrm{TC}_{it}$) is calculated as the ratio of accounts receivable ($\textrm{AR}_{it}$) to total sales ($\textrm{Sales}_{it}$), and controls for the length of time that the firm collects receivables from its customers. Second, the inventory period ($\textrm{INV}_{it}$) is measured as the ratio of monetary value of stocks ($I_{it}$) to the cost of goods sold ($\textrm{COGS}_{it}$), and captures the period during which the firm is able to sell their products. Third, the trade debit period ($\textrm{TD}_{it}$) is computed as the ratio of the accounts payable ($\textrm{AP}_{it}$) to $\textrm{COGS}_{it}$ and measures the period in which the firm pays its providers.

The CCC is a useful indicator for measuring the liquidity of a firm. More precisely, the CCC can be defined as the length of time between cash payments for the purchase of resalable goods and collection of accounts generated by sale of these goods. In other words, the CCC represents the time that a firm has invested in their working capital. Ideally, the firm is meant to shorten the CCC as much as possible without hurting operations. Otherwise, a longer CCC would increase the need for relatively costly external financing. Arithmetically, a positive (negative) sign of $\textrm{CCC}_{it}$ reveals that the company takes longer (shorter) to collect invoices from its customers and/or stock products than it does to pay its suppliers.^8^

**Fig. 1:**
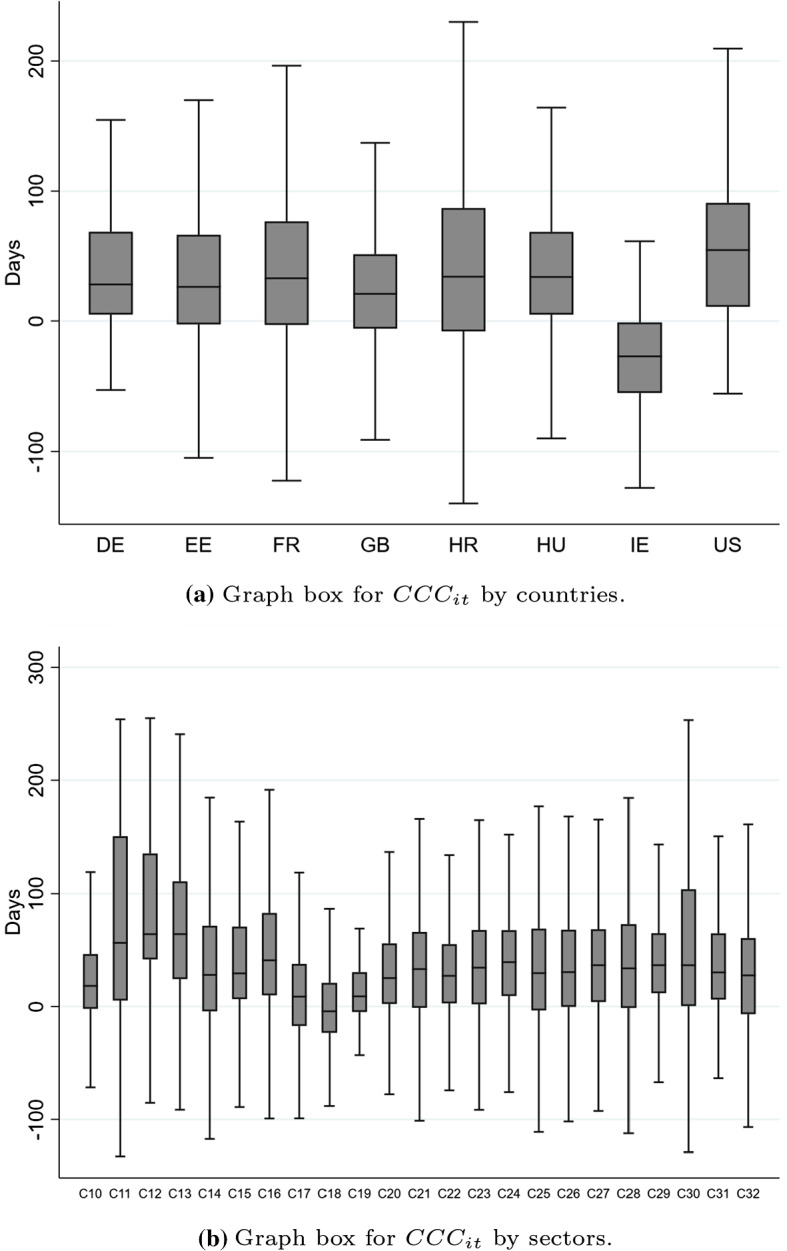
Distribution of cash conversion cycle ($\textrm{CCC}_{it}$) *Source:* Authors’ elaboration based on the Orbis database (*Bureau van Dijk*). The cash conversion cycle indicator ($\textrm{CCC}_{it}$) is defined in expression (1). Subfigure **a** is a box and whiskers plot of the distribution of $\textrm{CCC}_{it}$, distinguishing by the following countries: DE (Germany), EE (Estonia), FR (France), GB (United Kingdom), HR (Croatia), HU (Hungary), Ireland (IE) and US (United States). Subfigure **b** displays the box and whiskers plot of the distribution of $\textrm{CCC}_{it}$ distinguishing by NACE sectors as defined in Table 8 The whiskers show the upper and lower limits of the distribution. The line through each box indicates the median, i.e. the 50th percentile of the distribution. The upper (lower) boundaries of the box represent the 25th percent of the sample greater (lower) than the median, i.e. the upper (lower) quartile

Figure 1 represents the distribution of the CCC by countries and manufacturing sector, as covered by NACE Rev. Subfigure (a), which represents the box and whiskers graph of $\textrm{CCC}_{it}$ by countries, shows that the distributions display similar values of CCC. The chart suggests that the sample is not affected by country effects that might steer the subsequent econometric analysis. Interestingly, Subfigure (b), which illustrates the distribution of $\textrm{CCC}_{it}$ by NACE manufacturing sectors, reveals that there are no significant differences in the distributions between them. Nonetheless, a certain degree of dispersion can be observed among sectors when comparing the extreme values of the distributions.

### Financial constraint measures

This subsection discusses the indicators of financial constraints used in this article: (i) the Hadlock and Pierce (2010) SA index, (ii) the Whited and Wu (2006) index and (iii) the interest burden indicator (see Spaliara 2009; Chen and Guariglia 2013; Fernandes et al. 2019).

#### Hadlock and Pierce’s (2010) SA index

We compute the Hadlock and Pierce (2010) SA index ($\textrm{SA}_{it}$) as the primary measure of firms’ external finance constraints. This indicator is based on the notion that the largest and the oldest firms are comparatively more likely to borrow (see Collier et al. 2020). This indicator is calculated as follows:

$$\begin{aligned} \textrm{SA}_{it}=-0.737\times \textrm{Size}_{it}+0.043\times \textrm{Size}_{it}^{2}-0.040\times \textrm{Age}_{it} \end{aligned}$$

where $\textrm{Size}_{it}$ is the natural logarithm of a firm’s *i* total assets ($\textrm{TA}_{it}$) and controls for firm size. The age of the firm ($\textrm{Age}_{it}$) is calculated as the difference between the current period *t* and the year in which the firm was founded.

The SA index can be interpreted as the firm’s (inverse) probability of borrowing external resources.

Following Hirsch and Walz (2017), we consider a firm as financially constrained if it belongs to the highest part of the distribution of $\textrm{SA}_{it}$. Thus, we classify firms by constructing the dummy variable ($\textrm{SAD}_{it}$) which takes the value one if the firm *i* at period *t* belongs to centile ($\ge 1/3$) of the distribution, and zero otherwise.^9^

#### The Whited and Wu (2006) index

To test the robustness of the results obtained from the SA index, we calculate the Whited and Wu (2006) index ($\textrm{WW}_{it}$). This indicator is built on the basis of size and information about the financing structure of the firm. The WW index is calculated as follows:

$$\begin{aligned} \begin{aligned} \textrm{WW}_{it}&= -0.091\times \textrm{CFA}_{it}-0.062\times \textrm{DIVPOS}_{it}+0.021\times \textrm{TLTD}_{it} \\&\quad -0.044\times \textrm{Size}_{it}+0.102\times \textrm{ISG}_{kt}-0.035\times \textrm{SG}_{it} \end{aligned} \end{aligned}$$

where $\textrm{CFA}_{it}$ is the cash flow to total assets ($\textrm{TA}_{it}$) indicator and controls for business generation. The variable $\textrm{DIVPOS}_{it}$ is a dummy variable that takes value one if the growth rate of retained earnings between $t+1$ and *t* exceeds net income in year *t*, and zero otherwise. The variable $\textrm{TLTD}_{it}$ represents a firm’s indebtedness and is measured as the long-term debt-to-$\textrm{TA}_{it}$ ratio. The indicator $\textrm{Size}_{it}$ is defined in the previous section. The indicator $\textrm{ISG}_{kt}$ is the gross rate of gross production value at the industry level (*k*). Lastly, $\textrm{SG}_{it}$ is the firm’s sales growth.

As previously stated, firms belonging to the highest part of the distribution of $\textrm{WW}_{it}$ are defined as financially constrained. Accurately, the dummy $\textrm{WWD}_{it}$ takes value one if the firm belongs to centile ($\ge 1/3$), that is, if the firm is financially constrained, and $\textrm{WWD}_{it}=0$ when the firm is considered unconstrained.^10^

#### The interest burden indicator

We also include the interest burden indicator ($\textrm{IB}_{it}$) as an additional measure of financial constraints (Fernandes et al. 2019).^11^ This indicator is calculated as the interest-rates-payments-to-cash-flow ratio (see Benito and Hernando 2008; Nickell and Nicolitsas 1999). The IB indicator captures the weight of interest rate that the firm pays out due to banks’ credit tightening. The IB can be interpreted as the variation in the firm’s interest rates payments due to changes in its financial position. In other words, financially constrained firms usually display relatively higher values of $\textrm{IB}_{it}$ (Mulier et al. 2016).

We subsequently create the dummy variable $\textrm{IBD}_{it}$ that takes value one if the observation is included in at least the 2/3 centile of the distribution of the IB indicator, and zero otherwise, and break the sample down into financially constrained ($\textrm{IBD}_{it}=1$) and unconstrained ($\textrm{IBD}_{it}=0$) firms.

**Fig. 2:**
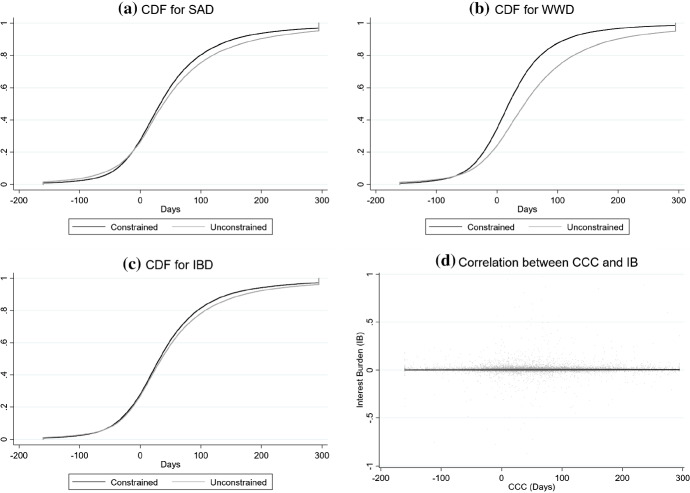
Cumulative distribution functions (CDF) of cash conversion cycle ($\textrm{CCC}_{it}$) by financial constraint indicators *Source:* Authors’ elaboration based on the Orbis database (*Bureau van Dijk*). The cash conversion cycle indicator ($\textrm{CCC}_{it}$) is defined in expression (1). Subfigure **a** represents the cumulative distribution function (CDF) of $\textrm{CCC}_{it}$ using the Whited and Wu (2006) index that classifies firms as financially constrained ($\textrm{SAD}_{it}=1$) and unconstrained ($\textrm{SAD}_{it}=0$). Subfigure **b** displays the CDF of of $\textrm{CCC}_{it}$ using the Hadlock and Pierce (2010) SA index that classifies firms as financially constrained ($\textrm{WWD}_{it}=1$) and unconstrained ($\textrm{WWD}_{it}=0$). Subfigure **c** shows the CDF of $\textrm{CCC}_{it}$ using the interest burden dummy ($\textrm{IBD}_{it}$) that classifies firms as financially constrained ($\textrm{IBD}_{it}=1$) and unconstrained ($\textrm{IBD}_{it}=0$). The interpretation of the CDF curve proceeds as follows. The horizontal axis represents observed values of $\textrm{CCC}_{it}$ in days, whereas the vertical axis shows the probability of finding each value. Subfigure **d** represents the correlation between $\textrm{CCC}_{it}$ (horizontal axis) and interest burden indicator ($\textrm{IB}_{it}$) (vertical axis). Each point represents a firm-year observation, whereas the black line fits the regression slope between both variables

Having discussed the aforementioned financial constraint indicators, Fig. 2 investigates whether these variables and $\textrm{CCC}_{it}$ can be considered uncorrelated variables.

Figure 2a–c represents the cumulative distribution function (CDF) of $\textrm{CCC}_{it}$, which splits the sample into financially constrained and unconstrained firms according to the above-discussed indicators ($\textrm{SAD}_{it}$, $\textrm{WWD}_{it}$, $\textrm{IB}_{it}$ and $\textrm{IBD}_{it}$). The overlapped distributions suggest that $\textrm{CCC}_{it}$ cannot be considered statistically different between financially constrained and unconstrained firms, regardless of the classification method. Importantly, Fig. 2d represents the zero-slope between $\textrm{CCC}_{it}$ (in the horizontal axis) and $\textrm{IB}_{it}$ (in the vertical axis), thus suggesting that both variables are uncorrelated.

### Baseline regression: Heckman’s estimator

This section presents the strategy to identify and to estimate the effects of working capital management on firms’ export behaviour. To do this, we follow the two-stage procedure of Heckman (1979) to address self-selection—or truncated selection—bias among exporters as follows. First, we estimate the impact of working capital management and financial constraints on firms’ decision to export. Second, we investigate how working capital management and financial constraints determine the volume of exports, taking into account that exporters are not a random subset of all firms, but may have characteristics that are also linked to how intense their export activity is (e.g. Máñez et al. 2008; Blanes-Cristóbal et al. 2008).

#### Firms’ decision to export: Selection equation

We closely follow the approach by Clerides et al. (1998) and Roberts and Tybout (1997) to modelling a multi-period export decision for entry in the export market in the presence of sunk costs. They consider that in each year *t*, a firm decides to export if the increment of the expected gross profits associated with exporting is positive. Importantly, sunk costs are the cornerstone for exporters since firms entering foreign markets have to engage in several activities related to exporting, e.g. market research, setting up an entrepreneurial network, adapting products, among others. Importantly, the aforementioned sunk costs are irreversible investments independent of the fact that firms continue exporting or not. Consequently, sunk costs related to current exports depend on whether the firm has exported previously. To take into account this dependence, we include in the selection equation—which models the likelihood of exporting—the export status in year $t-1$, and a matrix of variables that proxies for the pay-offs of exporting and firms’ capabilities to generate profits above sunk costs. Additionally, we also include working capital and financial constraint variables as defined in previous sections since they constitute the kernel of our research. A similar strategy has been used by Doan et al. (2020), Fauceglia (2015), Máñez and Vicente-Chirivella (2020) to capture the influence of financial constraints on exports, while Jinjarak and Wignaraja (2016) and Secchi et al. (2016) use other two-step approaches that are similar in spirit.

We propose the following selection equation of the decision to export:

2 $$\begin{aligned} ExpD_{it} = \left\{ \begin{array}{ll} 1 &{}\quad \text {if } \gamma _{0}ExpD_{i,t-1} + \gamma _{1}\textrm{CCC}_{i,t-1} + \gamma _{2}F_{i,t-1} + \gamma _{3}\textrm{CCC}_{i,t-1}\times F_{i,t-1} \\ &{}\quad + \,Z_{i,t-1}^{'}\Gamma + \nu _{k} + \tau _{t} + \omega _{it} \ge 0 \\ 0 &{}\quad \text {otherwise} \end{array}\right. \end{aligned}$$

where $ExpD_{it}$ is a dummy that takes value one if the firm *i* exports in year *t*, and zero otherwise, and represents the export status. $ExpD_{i,t-1}$ represents the export status in the previous year. Accordingly, the parameter $\gamma _{0}$ captures the influence of sunk costs of exporting. If significant, these coefficients should be interpreted as the rate of depreciation of export market experience and accumulated knowledge in foreign markets on the likelihood of exporting.

Regarding the explanatory variables, the indicator of interest is the one-period-lagged cash conversion cycle ($\textrm{CCC}_{i,t-1}$), which is computed as shown in expression (1). Furthermore, the variable $F_{i,t-1}$ takes value 1 if the firm was financially constrained the year before, according to one of the three financial constraint indicators described in Sect. 3.3. The matrix $Z_{i,t-1}^{'}$ includes the following vectors. Firm age ($\textrm{Ln}(\textrm{Age}_{i,t-1})$) is measured as the natural logarithm of $\textrm{Age}_{i,t-1}$ and controls for a firm’s experience. Nonlinearities in firm age is controlled by age-squared ($\textrm{Ln}(\textrm{Age}_{i,t-1})^{2}$). We control for firm size as the natural logarithm of the number of employees ($\textrm{Ln}(\textrm{Emp}_{i,t-1})$). Firm productivity $\left( Ln\left( \textrm{TFP}_{i,t-1}\right) \right)$ is computed as the natural logarithm of the total factor productivity, which is calculated following the Levinsohn and Petrin (2003) methodology. Importantly, $\textrm{Covid}_{t}$ is a dummy variable that takes value one if $t=2020$ and zero if $t<2020$ and controls for the COVID pandemic outbreak. Lastly, the variable $\nu _{k}$ corresponds to industry-fixed effects as listed in Table 8, $\tau _{t}$ are the year-fixed effects, and $\omega _{it}$ is the error term. The inclusion of time-specific effects aims to capture macro-level changes in export conditions like temporal variations in export profitability, start-up costs that are common across firms, the influence of business cycle, credit-market conditions, aggregate exchange rate movements, trade-policy conditions, overall changes in demand for Spanish exports and other time-varying factors. The industry dummies control for unobservable market characteristics where firms compete, being proxies of market concentration, use of technology or firms’ behaviour by industry.

#### The impact of CCC and financial constraints on exports

Modelling exports requires considering the possibility that exporters are not a random subset of all firms but may have characteristics that are also linked to their export activity. We tackle this problem using a two-stage sample selection procedure (Heckman 1979). From the estimation of the export participation (2), Heckman’s lambda ($\lambda _{it}$) is computed. A significant estimate for $\lambda _{it}$ in (2) would suggest the need to include it in the equation that determines the export volume to avoid a sample selection bias. In the second stage, we include this term as an additional regressor in the estimation of (3). The equation that assesses the impact of working capital management and financial constraints on firms’ volume of exports is the following:

3 $$\begin{aligned} \begin{aligned} \textrm{Ln}(E_{i,t})&= \alpha _{0} +\alpha _{1} \textrm{CCC}_{i,t-1}+\alpha _{2} F_{i,t-1} + \alpha _{3} \textrm{CCC}_{i,t-1}\times F_{i,t-1} \\&\quad + X_{i,t-1}^{'} \Psi +\nu _{k}+ \tau _{t} +\epsilon _{it} \end{aligned} \end{aligned}$$

where the dependent variable is the natural logarithm of export revenues ($E_{it}$).

Regarding the explanatory variables, the $X_{i,t-1}^{'}$ matrix adds the following variables to $Z_{i,t-1}^{'}$. Business generation ($\textrm{CFK}_{i,t-1}$) is measured as the cash-flow-to-fixed-assets ratio and controls for a firm’s capacity to generate wealth in the future. Leverage ($\textrm{LEV}_{i,t-1}$) is computed as the liabilities-to-equity ratio plus one and represents the level of risk that the firm is able to run.

To control for business cycle effects on a firm’s exports, we also include the GDP growth rate ($\textrm{GDP}_{ht}$) in the NUTS1 region h where the firm is headquartered.

**Table 2:** Descriptive statistics

Panel A: Description of the sample									Panel B: Differences in means		
									$$H_{0}: Exporters(1) - Exporters(0)$$		
	*N*	Mean	SD	Min.	Pc. 25	Median	Pc. 75	Max.	Exporter	Non-exporter	*p* value
*Dependent variables*											
$E_{it}$ (in EUR)	123,543	1,271,687	3,126,170	0.000	0.000	1.236	423,982	1.24E+07	–	–	–
$ExpD_{it}$	123,543	0.608	0.488	0.000	0.000	1.000	1.000	1.000	–	–	–
*Working capital management indicators*											
$\textrm{CCC}_{it}$	123,543	47.357	82.558	$-$ 136.992	$-$ 3.998	33.983	85.788	254.625	45.517	52.281	0.0000
$\textrm{TC}_{it}$	123,543	39.523	35.606	0.000	12.737	31.036	55.000	145.411	36.830	46.726	0.0000
$\textrm{INV}_{it}$	123,543	21.642	51.752	0.460	4.034	7.375	15.607	503.815	19.192	28.198	0.0000
$\textrm{TD}_{it}$	123,543	62.799	59.436	0.000	25.000	50.021	81.000	486.000	66.209	61.524	0.0000
*Control variables*											
$\textrm{Age}_{it}$	123,523	22.217	13.949	0.000	14.000	21.000	25.000	214.000	22.939	20.283	0.0000
$\textrm{Emp}_{it}$ (People)	123,543	47.643	83.457	1.000	4.000	12.000	44.000	334.000	23.166	67.644	0.0000
$\textrm{CFK}_{it}$	123,543	1.560	4.589	$-$ 1.586	0.154	0.368	0.978	37.043	1.566	1.545	0.4750
$\textrm{LEV}_{it}$	123,543	3.449	4.415	1.030	1.469	2.088	3.470	34.308	3.294	3.862	0.0000
$\textrm{Ln}(\textrm{TFP}_{it})$	123,543	9.414	0.988	4.584	8.702	9.349	9.995	15.570	9.505	9.171	0.0000
$\textrm{Covid}_{t}$	123,543	0.111	0.314	0.000	0.000	0.000	0.000	1.000	–	–	–
$\textrm{GDP}_{ht}$	123,543	1.469	2.852	$-$ 16.278	1.095	2.350	3.150	25.176	–	–	–
*Financial constraints indicators*											
$\textrm{SAD}_{it}$	123,543	0.240	0.124	0.000	0.000	0.000	0.000	1.000	0.170	0.262	0.0000
$\textrm{WWD}_{it}$	123,543	0.285	0.142	0.000	0.000	0.000	0.000	1.000	0.267	0.294	0.0000
$\textrm{IB}_{it}$	123,543	0.002	0.017	$-$ 0.870	0.000	0.001	0.002	0.971	0.001	0.003	0.0000
$\textrm{IBD}_{it}$	123,543	0.289	0.135	0.000	0.000	0.000	1.000	1.000	0.273	0.298	0.0000

This table reports the distribution of the variables used in this research between 2012 and 2020. Panel A shows the sample distribution for the whole period. Panel B displays the mean values of the same variables distinguishing between exporters and non-exporters. Parametric tests perform the statistical inference between sample means of the variables under the null of equality between both groups of firms ($H_{0}: Exporters(1) - Exporters(0)$). This sample includes companies from Croatia, Estonia, France, Germany, Hungary, Ireland, the UK and the USA during each period. Monetary values were adjusted by the inflation rate. All firm variables are winsorized at the 1% level to remove outliers. All the variables are defined in Table 1

Lastly, the variables $\nu _{k}$ and $\tau _{t}$ correspond to industry- and year-fixed effects, respectively. Importantly, there might be unobserved factors of the firms that might affect their export capacity such as product quality, managerial skills, or personnel abilities (see Máñez and Vicente-Chirivella 2020). Consequently, we assume that the error term $\epsilon _{it}$ has two components: the firm-specific effect ($\alpha _{i}$) and the transitory component ($u_{it}$), and then, $\epsilon _{it} = \alpha _{i} + u_{it}$.^12^

## Results

### Descriptive statistics and univariate analysis

Table 2 reports summary statistics for the whole sample of firms included in this study. The values of Panel A confirm the absence of outliers in the sample. The results of the parametric test of means are shown in Panel B. As a first step, we break the sample down between exporters (*Exporters*(1)) and non-exporters (*Exporters*(0)). The alternative hypothesis is confirmed for the cash conversion cycle indicator ($\textrm{CCC}_{it}$) ($H_{0}: Exporters(1)-Exporters(0)<0$), thus indicating that exporters enjoy comparatively shorter CCC periods in comparison with non-exporters. Disentangling $\textrm{CCC}_{it}$, the parametric test also rejects the null for the trade credit period ($\textrm{TC}_{it}$), thus indicating that exporters usually collect their bills comparatively sooner than non-exporters ($H_{0}: Exporters(1)-Exporters(0)<0$). Similarly, the parametric test also rejects the null for inventory period ($\textrm{INV}_{it}$) indicators, thus suggesting that exporters are able to circulate their stocks comparatively faster than non-exporters ($H_{0}: Exporters(1)-Exporters(0)<0$). On the other hand, the test also suggests that the trade debit period ($\textrm{TD}_{it}$) is relatively longer for exporters than for non-exporters ($H_{0}: Exporters(1)-Exporters(0)>0$), thus suggesting that exporters are comparatively more able to delay payments to providers. As proposed in Hypothesis 1, we find that exporters enjoy relatively shorter periods of CCC than non-exporters. Importantly, regarding the financial constraint indicators, the parametric test rejects the null for the three financial constraint dummy variables $\left( F_{it}= \left\{ \textrm{SAD}_{it}, \textrm{WWD}_{it}, \textrm{IBD}_{it} \right\} \right)$, thus revealing that the proportion of exporters classified as financially constrained is comparatively lower than non-exporters.

**Fig. 3:**
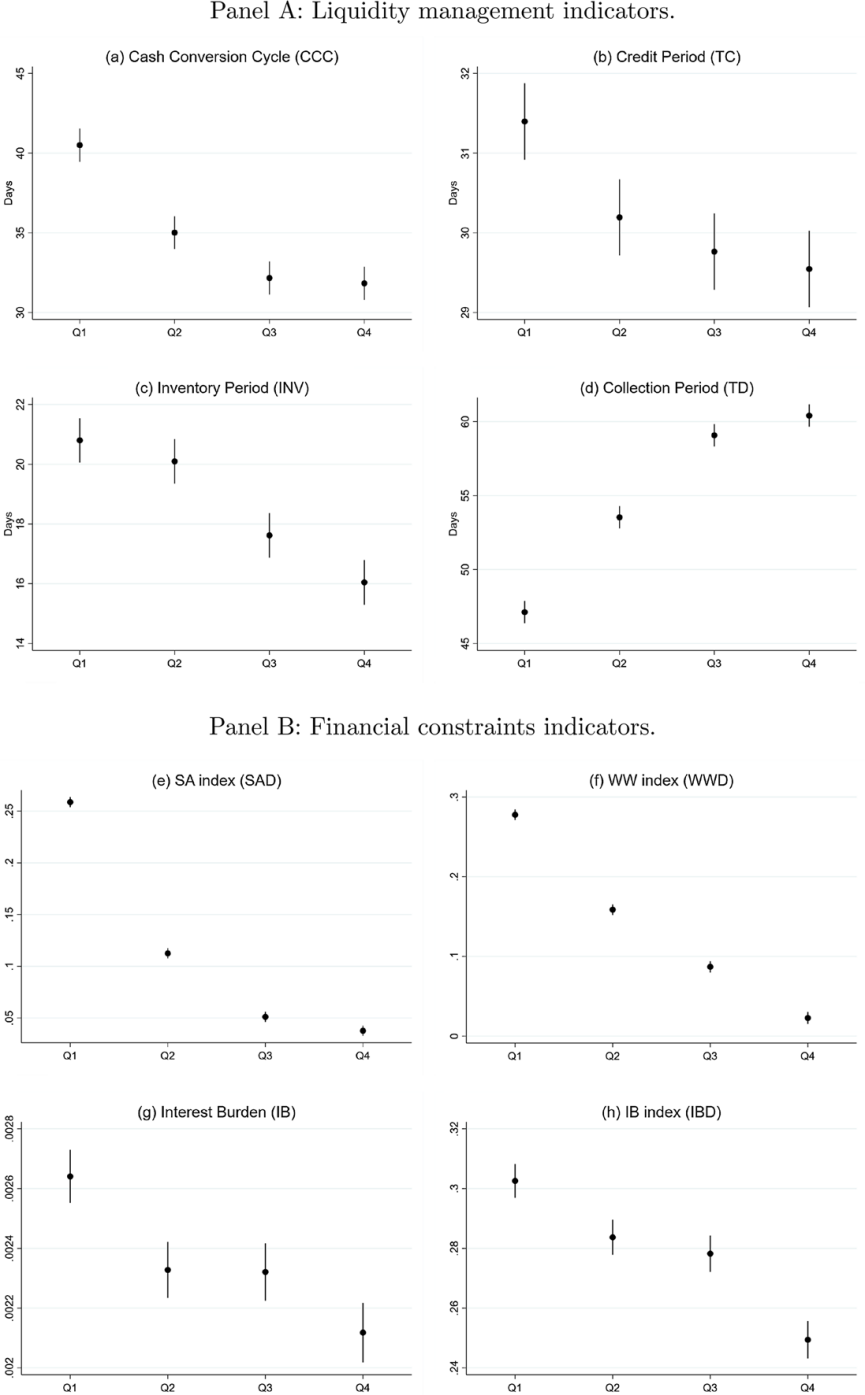
Statistics of the key variables depending on the quartiles of $\textrm{Ln}(E_{it})$ This figure decomposes the descriptive statistics of the key variables depending on the quartiles of exports ($\textrm{Ln}(E_{it})$) between 2012 and 2020. Annual observations for British, Croatian, Estonian, French, German, Hungarian, Irish and US firms are applied from 2012 to 2020. In Panel A, the dependent variables are the cash conversion cycle ($\textrm{CCC}_{it}$), the trade credit cycle ($\textrm{TC}_{it}$), the inventories cycle ($\textrm{INV}_{it}$), and the trade debit cycle ($\textrm{TD}_{it}$). In Panel B, the dependent variables are the following financial constraint indicators: the Hadlock and Pierce (2010) SA index ($\textrm{SAD}_{it}$), the Whited and Wu (2006) index ($\textrm{WWD}_{it}$), and the interest-paid-to-cash-flow ratio ($\textrm{IB}_{it}$), and the above 1/3-percentile of $\textrm{IB}_{it}$ ($\textrm{IBD}_{it}$). The estimation is conducted using the ordinary least squared (OLS) estimator. The regression coefficients represent the mean value of each dependent variable, whereas the bars represent the standard errors. The firm variables are winsorized at the 1% level to remove outliers. All the variables are defined in Table 1

In the light of the previous assessment, we now look for linear trends of the working capital management and financial constraint indicators as export revenues accumulate. For this purpose, Fig. 3 represents mean values and standard errors of the key variables, breaking the sample down into quartiles of the natural logarithm of exports ($\textrm{Ln}(E_{it})$). Panel A presents the estimates of $\textrm{Ln}(E_{it})$ on the working capital management indicators of this study. The results confirm that the higher the export sales ($\textrm{Ln}(E_{it})$), the lower the cash conversion cycle ($\textrm{CCC}_{it}$), thus indicating that exporters are able to reduce the length of time that firms’ cash is tied up within business operations. Interestingly, the trade credit period ($\textrm{TC}_{it}$) and the inventory period ($\textrm{INV}_{it}$) decrease with exports, thus reflecting the effects of the opportunity cost of granting credit to customers and stocking in warehouses. Trade debit period ($\textrm{TD}_{it}$) consistently displays an ascending trend on $\textrm{Ln}(E_{it})$, thus confirming that exporters have comparatively better access to trade finance than non-exporters. Panel B displays the estimates on the financial constraint indicators. The interest burden ($\textrm{IB}_{it}$) shows a decreasing trend on exports, which reveals that exporters face less pressure from interest payments. Accordingly, estimates on the financial constraint classifiers $\left( F_{it}\right)$ suggest that financially constrained firms tend to concentrate in the lowest quartiles of $\textrm{Ln}(E_{it})$. Taken altogether, the results suggest that exporters are less likely to face financial constraints than non-exporters.

Overall, the results discussed in this section confirm prior findings that exporters have generally better access to external finance—i.e. being less likely to be financially constrained—than non-exporters (e.g. Muûls 2015; Minetti and Zhu 2011; Minetti et al. 2018). Furthermore, we find that exporters display comparatively lower values of CCC, which might be a symptom that these firms manage their liquidity more efficiently and need less short-term financing (see Deloof 2003; Wang 2019; Zeidan and Shapir 2017), as proposed by Hypothesis 1.

**Fig. 4:**
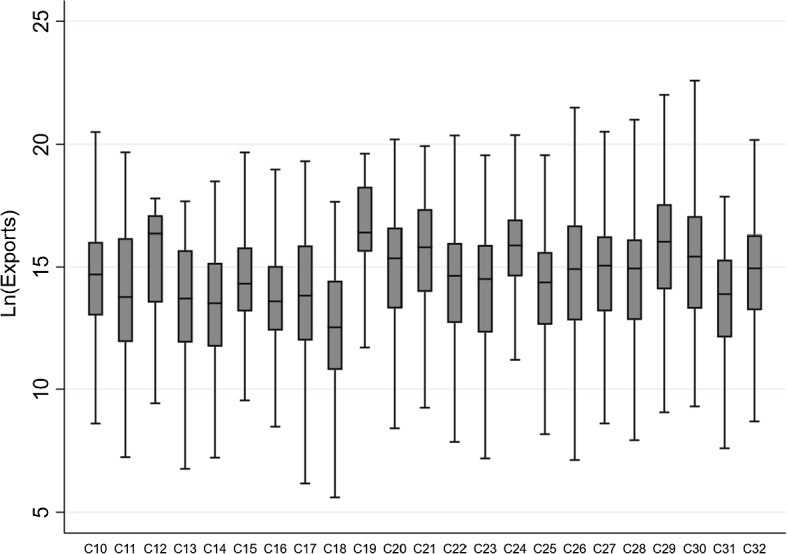
Distribution of $\textrm{Ln}(E_{it})$ by sectors *Source:* Authors’ elaboration based on the Orbis database (*Bureau van Dijk*). The figure represents the box and whiskers plot of the distribution of the natural logarithm of the volume of exports ($\textrm{Ln}(E_{it})$) as defined in Table 1, distinguishing by sectors. The horizontal axis displays the following NACE sectors: C-Manufacturing, D-Electricity, gas, steam and air conditioning supply; E-Water supply; sewerage, waste management and remediation activities; F-Construction; G-Wholesale and retail trade; repair of motor vehicles and motorcycles; H-Transportation and storage. The whiskers show the upper and lower limits of the distribution. The line through each box indicates the median, i.e. the 50th percentile of the distribution. The upper (lower) boundaries of the box represents the 25th percent of the sample greater (lower) than the median, i.e. the upper (lower) quartile

Last but not least, we now look for variability among industry sectors in terms of exports. Figure 4 shows the distribution of $\textrm{Ln}(E_{it})$, distinguishing between the sectors included in our sample. The overlapped distributions suggest that there are no significant differences in $\textrm{Ln}(E_{it})$ between industries. However, a certain degree of variability can be witnessed in the extreme values of the distributions, which might justify the inclusion of industry fixed effects ($\nu _{k}$) in the regression analysis.

### Baseline results

This section discusses the econometric results that test the hypotheses of this research. Table 3 displays the regression coefficients for the Heckman (1979) estimator.

Panel A displays the regression results for the **selection equation** as shown in expression (2). Estimates on $ExpD_{i,t-1}$ are positive and significant revealing that entry barriers are crucial in firms’ internationalization processes. Importantly, estimates on the cash conversion cycle variable ($\textrm{CCC}_{i,t-1}$) are negative and significant, which indicates that relatively longer working capital cycles reduce the probability of deciding to enter foreign markets. This result confirms Hypothesis 1 in terms of becoming exporters. Additionally, estimates on financial constraint indicators ($F_{i,t-1}$) are negative and significant, which indicate that the lack of access to financial resources reduces the probability of selling abroad. Importantly, estimates on the interactions between the cash conversion cycle and financial constraint indicators ($\textrm{CCC}_{i,t-1} \times F_{i,t-1}$) are negative and significant, which suggests that the impact of relatively longer working capital cycles is worsened if the firm is unable to obtain external funding. These results corroborate Hypothesis 2, which proposes that financial constraints heightens the impact of CCC on firms’ exports. Regarding the other explanatory variables, all the estimates display the expected signs and level of significance.

**Table 3:** The effects of liquidity management and financial constraints on exports. Baseline results

	$\textrm{SAD}_{i,t-1}$		$\textrm{WWD}_{i,t-1}$		$\textrm{IBD}_{i,t-1}$	
	(1)	(2)	(3)	(4)	(5)	(6)
*Panel A: Export decision (average marginal effects)*						
$$ExpD_{i,t-1}$$	0.325$$^{***}$$	0.325$$^{***}$$	0.355$$^{***}$$	0.355$$^{***}$$	0.335$$^{***}$$	0.335$$^{***}$$
	(0.003)	(0.003)	(0.002)	(0.002)	(0.002)	(0.002)
$$\textrm{CCC}_{i,t-1}$$	$$-$$ 0.001$$^{***}$$	$$-$$ 0.001$$^{***}$$	$$-$$ 0.001$$^{***}$$	$$-$$ 0.001$$^{***}$$	$$-$$ 0.001$$^{***}$$	$$-$$ 0.001$$^{***}$$
	(0.000)	(0.000)	(0.000)	(0.000)	(0.000)	(0.000)
$$F_{i,t-1}$$	$$-$$ 0.013$$^{***}$$	$$-$$ 0.013$$^{***}$$	$$-$$ 0.011$$^{***}$$	$$-$$ 0.011$$^{***}$$	$$-$$ 0.016$$^{***}$$	$$-$$ 0.016$$^{***}$$
	(0.002)	(0.002)	(0.002)	(0.002)	(0.002)	(0.002)
$$\textrm{CCC}_{i,t-1} \times F_{i,t-1}$$		$$-$$ 0.001$$^{***}$$		$$-$$ 0.001$$^{***}$$		$$-$$ 0.001$$^{***}$$
		(0.000)		(0.000)		(0.000)
$$\textrm{Ln}(\textrm{Age}_{i,t-1})$$	0.024$$^{***}$$	0.024$$^{***}$$	0.027$$^{***}$$	0.027$$^{***}$$	0.024$$^{***}$$	0.024$$^{***}$$
	(0.002)	(0.002)	(0.002)	(0.002)	(0.002)	(0.002)
$$\textrm{Ln}(\textrm{Age}_{i,t-1})^{2}$$	$$-$$ 0.007$$^{***}$$	$$-$$ 0.007$$^{***}$$	$$-$$ 0.007$$^{***}$$	$$-$$ 0.007$$^{***}$$	$$-$$ 0.007$$^{***}$$	$$-$$ 0.007$$^{***}$$
	(0.001)	(0.001)	(0.002)	(0.002)	(0.001)	(0.001)
$$\textrm{Ln}(\textrm{Emp}_{i,t-1})$$	0.014$$^{***}$$	0.014$$^{***}$$	0.016$$^{***}$$	0.016$$^{***}$$	0.020$$^{***}$$	0.020$$^{***}$$
	(0.001)	(0.001)	(0.001)	(0.001)	(0.001)	(0.001)
$$\textrm{Ln}(\textrm{TFP}_{i,t-1})$$	0.013$$^{***}$$	0.013$$^{***}$$	0.011$$^{***}$$	0.011$$^{***}$$	0.016$$^{***}$$	0.016$$^{***}$$
	(0.002)	(0.002)	(0.001)	(0.001)	(0.001)	(0.001)
$$\textrm{Covid}_{t}$$	$$-$$ 0.007$$^{***}$$	$$-$$ 0.007$$^{***}$$	$$-$$ 0.011$$^{***}$$	$$-$$ 0.011$$^{***}$$	$$-$$ 0.008$$^{***}$$	$$-$$ 0.008$$^{***}$$
	(0.002)	(0.002)	(0.002)	(0.002)	(0.002)	(0.002)
Industry FE	Yes	Yes	Yes	Yes	Yes	Yes
Year FE	Yes	Yes	Yes	Yes	Yes	Yes
*Panel B: Export volume*						
$$\textrm{CCC}_{i,t-1}$$	$$-$$ 0.001$$^{***}$$	$$-$$ 0.001$$^{***}$$	$$-$$ 0.001$$^{***}$$	$$-$$ 0.001$$^{***}$$	$$-$$ 0.001$$^{***}$$	$$-$$ 0.001$$^{***}$$
	(0.000)	(0.000)	(0.000)	(0.000)	(0.000)	(0.000)
$$F_{i,t-1}$$	$$-$$ 0.189$$^{***}$$	$$-$$ 0.189$$^{***}$$	$$-$$ 0.190$$^{***}$$	$$-$$ 0.191$$^{***}$$	$$-$$ 0.171$$^{***}$$	$$-$$ 0.171$$^{***}$$
	(0.027)	(0.028)	(0.027)	(0.027)	(0.014)	(0.014)
$$\textrm{CCC}_{i,t-1} \times F_{i,t-1}$$		$$-$$ 0.001$$^{***}$$		$$-$$ 0.001$$^{***}$$		$$-$$ 0.002$$^{***}$$
		(0.000)		(0.000)		(0.000)
$$\textrm{Ln}(\textrm{Age}_{i,t-1})$$	0.577$$^{***}$$	0.747$$^{***}$$	0.584$$^{***}$$	0.582$$^{***}$$	0.620$$^{***}$$	0.622$$^{***}$$
	(0.054)	(0.070)	(0.085)	(0.085)	(0.062)	(0.062)
$$\textrm{Ln}(\textrm{Age}_{i,t-1})^{2}$$	$$-$$ 0.082$$^{***}$$	$$-$$ 0.105$$^{***}$$	$$-$$ 0.074$$^{***}$$	$$-$$ 0.073$$^{***}$$	$$-$$ 0.085$$^{***}$$	$$-$$ 0.085$$^{***}$$
	(0.010)	(0.012)	(0.015)	(0.015)	(0.011)	(0.011)
$$\textrm{Ln}(\textrm{Emp}_{i,t-1})$$	0.543$$^{***}$$	1.056$$^{***}$$	0.592$$^{***}$$	0.592$$^{***}$$	0.608$$^{***}$$	0.608$$^{***}$$
	(0.005)	(0.006)	(0.007)	(0.007)	(0.006)	(0.006)
$$\textrm{CFK}_{i,t-1}$$	0.011$$^{***}$$	0.017$$^{***}$$	0.013$$^{***}$$	0.013$$^{***}$$	0.009$$^{***}$$	0.009$$^{***}$$
	(0.001)	(0.002)	(0.002)	(0.002)	(0.002)	(0.002)
$$\textrm{LEV}_{i,t-1}$$	$$-$$ 0.017$$^{***}$$	$$-$$ 0.017$$^{***}$$	$$-$$ 0.014$$^{***}$$	$$-$$ 0.014$$^{***}$$	$$-$$ 0.019$$^{***}$$	$$-$$ 0.019$$^{***}$$
	(0.002)	(0.002)	(0.002)	(0.002)	(0.002)	(0.002)
$$\textrm{Ln}(\textrm{TFP}_{i,t-1})$$	0.575$$^{***}$$	0.616$$^{***}$$	0.625$$^{***}$$	0.625$$^{***}$$	0.617$$^{***}$$	0.617$$^{***}$$
	(0.008)	(0.010)	(0.010)	(0.010)	(0.009)	(0.009)
$$\textrm{Covid}_{t}$$	$$-$$ 0.083$$^{***}$$	$$-$$ 0.083$$^{***}$$	$$-$$ 0.086$$^{***}$$	$$-$$ 0.086$$^{***}$$	$$-$$ 0.083$$^{***}$$	$$-$$ 0.083$$^{***}$$
	(0.016)	(0.016)	(0.016)	(0.016)	(0.016)	(0.016)
$$\textrm{GDP}_{h,t-1}$$	0.076$$^{***}$$	0.076$$^{***}$$	0.138$$^{***}$$	0.138$$^{***}$$	0.062$$^{***}$$	0.061$$^{***}$$
	(0.005)	(0.005)	(0.008)	(0.008)	(0.006)	(0.006)
$$\lambda _{it}$$	0.922$$^{***}$$	0.922$$^{***}$$	0.922$$^{***}$$	0.922$$^{***}$$	0.961$$^{***}$$	0.961$$^{***}$$
	(0.009)	(0.009)	(0.012)	(0.012)	(0.012)	(0.012)
Industry FE	Yes	Yes	Yes	Yes	Yes	Yes
Year FE	Yes	Yes	Yes	Yes	Yes	Yes
N	109,794	109,794	109,794	109,794	109,794	109,794
Wald’s test [*p* values]	0.000	0.000	0.000	0.000	0.000	0.000
LR test [*p* values]	0.000	0.000	0.000	0.000	0.000	0.000

This table reports the results of the impact of the cash conversion cycle ($$\textrm{CCC}_{i,t}$$) on firms’ exports. Annual observations for British, Croatian, Estonian, French, German, Hungarian, Irish and US firms are applied from 2012 to 2020. The dependent variable is the natural logarithm of exports ($$\textrm{Ln}(E_{it})$$). We break the sample down into financially constrained and unconstrained firms using the three following indicators ($$F_{i,t-1}$$): (i) $$\textrm{SAD}_{i,t-1}$$ is a dummy variable which equals one for observations with the Hadlock and Pierce (2010) SA index above the 1/3 percentile, and zero otherwise; (ii) $$\textrm{WWD}_{i,t-1}$$ is a dummy variable which equals one for observations with the Whited and Wu (2006) index above the 1/3 percentile, and zero otherwise; and the $$\textrm{IBD}_{i,t-1}$$ indicator is a dummy variable which equals one for observation with the interest-paid-to-cash-flow ratio ($$\textrm{IB}_{it}$$) above the 1/3 percentile, and zero otherwise. The matrix $$Z_{i,t-1}^{'}$$ includes the following control variables: $$\textrm{Ln}(\textrm{Age}_{i,t-1})$$, $$\textrm{Ln}(\textrm{Age}_{i,t-1})^{2}$$, $$\textrm{Ln}(\textrm{Emp}_{i,t-1})$$, $$\textrm{Ln}(\textrm{TFP}_{i,t-1})$$, $$\textrm{Covid}_{t}$$. The matrix $$X_{i,t-1}^{'}$$ adds to $$Z_{i,t-1}^{'}$$ the following variables: $$\textrm{CFK}_{i,t-1}$$, $$\textrm{LEV}_{i,t-1}$$, $$\textrm{GDP}_{h,t-1}$$. All the variables are defined in Table 1. The estimations are conducted using the Heckman (1979) estimator. Goodness of fit is tested using the Wald test (*p* values). Independence between the regression and the selection equations is tested using the LR test (*p* values) under the null of no selection process ($$H_{0}: \rho =0$$). Robust standard errors are presented in parentheses (White 1980). Estimates followed by $$^{*}$$, $$^{**}$$, $$^{***}$$ are statistically significant at the 10%, 5% and 1% levels, respectively

Panel B shows the regression coefficients for expression (3) explaining export volume. A common result to all specification is that the estimate corresponding to the Heckman’s lambda is positive and significant. First, this confirms the need of including this lambda in the export volume equation to avoid a sample selection bias; second, the positive sign suggests that the unobservables affecting firms’ likelihood of exporting are positively correlated with their export intensity. Importantly, the results clearly support our first hypothesis, according to which working capital financing plays a crucial role in the finance-export transmission channel. The estimates show that a 1% increase in the cash conversion cycle ($\textrm{CCC}_{it}$) reduces firms’ volume of exports by 0.001%, which indicates that relatively large liquidity cycles tend to penalize foreign sales. Furthermore, we test Hypothesis 2 by interacting the CCC and financial constraint indicators ($\textrm{CCC}_{i,t-1}\times F_{i,t-1}$). The estimated coefficients are negative and statistically significant, demonstrating that financially constrained exporters rely comparatively more on internal finance than unconstrained ones. These findings have relevant implications, particularly in times of crisis. The results of this study suggest that the higher the length of time in the production process, the lower firms’ capacity to export. Indeed, high levels of CCC might indicate firms’ need for external finance for working capital and reliance on short-term debt. Consequently, if firms are unable to raise funds, they can have more exposure to aggregate funding risk and might perform worse during crisis periods.^13^

**Table 4:** The effects of the components of CCC and financial constraints on exports

	$\textrm{TC}_{i,t-1}$			$\textrm{INV}_{i,t-1}$			$\textrm{TD}_{i,t-1}$		
	$\textrm{SAD}_{i,t-1}$	$\textrm{WWD}_{i,t-1}$	$\textrm{IBD}_{i,t-1}$	$\textrm{SAD}_{i,t-1}$	$\textrm{WWD}_{i,t-1}$	$\textrm{IBD}_{i,t-1}$	$\textrm{SAD}_{i,t-1}$	$\textrm{WWD}_{i,t-1}$	$\textrm{IBD}_{i,t-1}$
	(1)	(2)	(3)	(4)	(5)	(6)	(7)	(8)	(9)
*Panel A: Export decision (average marginal effects)*									
$$ExpD_{i,t-1}$$	0.320$$^{***}$$	0.323$$^{***}$$	0.324$$^{***}$$	0.320$$^{***}$$	0.323$$^{***}$$	0.324$$^{***}$$	0.320$$^{***}$$	0.323$$^{***}$$	0.324$$^{***}$$
	(0.001)	(0.001)	(0.001)	(0.001)	(0.001)	(0.001)	(0.001)	(0.001)	(0.001)
$$I_{i,t-1}$$	$$-$$ 0.002$$^{***}$$	$$-$$ 0.001$$^{***}$$	$$-$$ 0.002$$^{***}$$	$$-$$ 0.002$$^{***}$$	$$-$$ 0.001$$^{***}$$	$$-$$ 0.002$$^{***}$$	$$-$$ 0.002$$^{***}$$	$$-$$ 0.001$$^{***}$$	$$-$$ 0.002$$^{***}$$
	(0.000)	(0.000)	(0.000)	(0.000)	(0.000)	(0.000)	(0.000)	(0.000)	(0.000)
$$F_{i,t-1}$$	$$-$$ 0.027$$^{***}$$	$$-$$ 0.014$$^{***}$$	0.026$$^{***}$$	$$-$$ 0.027$$^{***}$$	$$-$$ 0.014$$^{***}$$	0.026$$^{***}$$	$$-$$ 0.027$$^{***}$$	$$-$$ 0.014$$^{***}$$	0.026$$^{***}$$
	(0.003)	(0.002)	(0.002)	(0.003)	(0.002)	(0.002)	(0.024)	(0.002)	(0.002)
$$I_{i,t-1} \times F_{i,t-1}$$	$$-$$ 0.002$$^{***}$$	$$-$$ 0.001$$^{***}$$	$$-$$ 0.001$$^{***}$$	$$-$$ 0.002$$^{***}$$	$$-$$ 0.002$$^{***}$$	$$-$$ 0.001$$^{***}$$	$$-$$ 0.002$$^{***}$$	$$-$$ 0.001$$^{***}$$	$$-$$ 0.001$$^{***}$$
	(0.000)	(0.000)	(0.000)	(0.000)	(0.000)	(0.000)	(0.000)	(0.000)	(0.000)
$Z_{i,t-1}^{'}$	Yes	Yes	Yes	Yes	Yes	Yes	Yes	Yes	Yes
Industry FE	Yes	Yes	Yes	Yes	Yes	Yes	Yes	Yes	Yes
Year FE	Yes	Yes	Yes	Yes	Yes	Yes	Yes	Yes	Yes
*Panel B: Export volume*									
$$I_{i,t-1}$$	$$-$$ 0.002$$^{***}$$	$$-$$ 0.002$$^{***}$$	$$-$$ 0.001$$^{***}$$	$$-$$ 0.001$$^{***}$$	$$-$$ 0.001$$^{***}$$	$$-$$ 0.001$$^{***}$$	0.001$$^{***}$$	0.001$$^{***}$$	0.001$$^{***}$$
	(0.000)	(0.000)	(0.000)	(0.000)	(0.000)	(0.000)	(0.000)	(0.000)	(0.000)
$$F_{i,t-1}$$	$$-$$ 0.255$$^{***}$$	$$-$$ 0.200$$^{***}$$	$$-$$ 0.239$$^{***}$$	$$-$$ 0.186$$^{***}$$	$$-$$ 0.189$$^{***}$$	$$-$$ 0.187$$^{***}$$	$$-$$ 0.242$$^{***}$$	$$-$$ 0.170$$^{***}$$	$$-$$ 0.201$$^{***}$$
	(0.026)	(0.031)	(0.022)	(0.021)	(0.021)	(0.021)	(0.027)	(0.031)	(0.022)
$$I_{i,t-1} \times F_{i,t-1}$$	$$-$$ 0.002$$^{***}$$	$$-$$ 0.002$$^{***}$$	$$-$$ 0.002$$^{***}$$	$$-$$ 0.001$$^{***}$$	$$-$$ 0.001$$^{***}$$	$$-$$ 0.001$$^{***}$$	0.001$$^{***}$$	0.001$$^{***}$$	0.001$$^{***}$$
	(0.001)	(0.001)	(0.001)	(0.000)	(0.000)	(0.000)	(0.000)	(0.000)	(0.000)
$$\lambda _{it}$$	0.920$$^{***}$$	0.991$$^{***}$$	0.962$$^{***}$$	0.920$$^{***}$$	0.991$$^{***}$$	0.962$$^{***}$$	0.920$$^{***}$$	0.991$$^{***}$$	0.962$$^{***}$$
	(0.009)	(0.012)	(0.011)	(0.009)	(0.012)	(0.011)	(0.009)	(0.012)	(0.011)
$X_{i,t-1}^{'}$	Yes	Yes	Yes	Yes	Yes	Yes	Yes	Yes	Yes
Industry FE	Yes	Yes	Yes	Yes	Yes	Yes	Yes	Yes	Yes
Year FE	Yes	Yes	Yes	Yes	Yes	Yes	Yes	Yes	Yes
N	109,794	109,794	109,794	109,794	109,794	109,794	109,794	109,794	109,794
Wald’s test [*p* values]	0.000	0.000	0.000	0.000	0.000	0.000	0.000	0.000	0.000
LR test [*p* values]	0.000	0.000	0.000	0.000	0.000	0.000	0.000	0.000	0.000

This table reports the results of the impact of the component of the cash conversion cycle on firms’ exports. Annual observations for British, Croatian, Estonian, French, German, Hungarian, Irish and US firms are applied from 2012 to 2020. The dependent variable is the natural logarithm of exports ($$\textrm{Ln}(1+E_{it})$$). $$I_{it}$$ refers to the credit period ($$\textrm{TC}_{it}$$) (Columns (1)–(3)), the inventory period ($$\textrm{INV}_{it}$$) (Columns (4)–(6)) and the collection period ($$\textrm{TD}_{it}$$) (Columns (7)–(9)). We break the sample down into financially constrained and unconstrained firms using the three following indicators ($$F_{i,t-1}$$): (i) $$\textrm{SAD}_{i,t-1}$$ is a dummy variable which equals one for observations with the Hadlock and Pierce (2010) SA index above the 1/3 percentile, and zero otherwise; (ii) $$\textrm{WWD}_{i,t-1}$$ is a dummy variable which equals one for observations with the Whited and Wu (2006) index above the 1/3 percentile, and zero otherwise; and the $$\textrm{IBD}_{i,t-1}$$ indicator is a dummy variable which equals one for observation with the interest-paid-to-cash-flow ratio ($$\textrm{IB}_{i,t-1}$$) above the 1/3 percentile, and zero otherwise. The matrix $$Z_{i,t-1}^{'}$$ includes the following control variables: $$\textrm{Ln}(\textrm{Age}_{i,t-1})$$, $$\textrm{Ln}(\textrm{Age}_{i,t-1})^{2}$$, $$\textrm{Ln}(\textrm{Emp}_{i,t-1})$$, $$\textrm{Ln}(\textrm{TFP}_{i,t-1})$$, $$\textrm{Covid}_{t}$$. The matrix $$X_{i,t-1}^{'}$$ adds to $$Z_{i,t-1}^{'}$$ the following variables: $$\textrm{CFK}_{i,t-1}$$, $$\textrm{LEV}_{i,t-1}$$, $$\textrm{GDP}_{h,t-1}$$. All the variables are defined in Table 1. The estimations are conducted using the Heckman (1979) estimator. Goodness of fit is tested using the Wald test (*p* values). Independence between the regression and the selection equations is tested using the LR test (*p* values) under the null of no selection process ($$H_{0}: \rho =0$$). Robust standard errors are presented in parentheses (White 1980). Estimates followed by $$^{*}$$, $$^{**}$$, $$^{***}$$ are statistically significant at the 10%, 5% and 1% levels, respectively

We disentangle the CCC indicator so as to investigate the role of each component on the dependent variable as shown in expression (1). Regarding the selection equations (Panel A of Table 4), all the signs and level of significance remain qualitatively similar to those presented above. Panel B displays the estimates for the equation for exports’ amounts. On the current assets side, columns (1)–(3) substitute $\textrm{CCC}_{i,t-1}$ with trade credit period ($\textrm{TC}_{i,t-1}$) as an independent variable to investigate the effects of granting [lengthy] trade credit on firms’ exports. The negative and statistically significant estimates on $\textrm{TC}_{i,t-1}$ suggest that the trade credit period reduces firms’ export revenues. This finding is in line with previous studies arguing that granting trade credit might generate opportunity costs for trade borrowers, because the former might renounce investing in specific assets, which might be needed to export, in favour of their customers. Interestingly, the estimates on $\textrm{TC}_{i,t-1} \times F_{i,t-1}$ also suggest that financial constraints also contribute to exacerbating the negative impact of trade credit on firms’ exports. In other words, credit-constrained firms are comparatively more exposed to customers’ solvency, which makes them vulnerable to aggregate risk (Raddatz 2006; Tong and Wei 2011; Wang 2019). Indeed, we find qualitatively similar results when introducing the inventory period indicator ($\textrm{INV}_{i,t-1}$) as a regressor (columns (4)–(6)). The negative and statistically significant estimates suggest that carrying and storage reduce the volume of firms’ sales. This result lends strong support to the hypothesis that a relatively inefficient management of inventories, or even a weak external demand—i.e. aggregate risk—exerts a deleterious effect on firms’ capacity to export. Remarkably, this impact is found to be economically more relevant for credit-constrained firms. The results do not necessarily contradict Deloof (2003), who predicted that trade credit fosters sales, since overcoming the above-mentioned opportunity costs is vital for exporters. On the current liabilities side, columns (7)–(9) display the estimates on the trade debit period ($\textrm{TD}_{i,t-1}$), which are positive and significant. Delays in payments may be a flexible source of financing for exporters, which enable them to reduce their cost structure (see Long et al. 1993), thus improving their capacity to sell abroad.

### Robustness checks

One concern with the interpretation of previous results might be that they could be affected by potential endogeneity of the export decision or misidentification issues. Thus, firms’ self-selection into export markets might be steered by unobservable factors correlated with the CCC or with credit constraints. Even if previous specifications are carried out using the Heckman (1979) estimator, which includes the selection equation to mitigate the so-called sample selection bias, this section performs additional tests to address the above-mentioned endogeneity concerns. Firstly, the propensity score matching investigates systematic differences between exporters and non-exporters. Secondly, we carry out a transition sample analysis to test whether ‘transitioning’ from constrained to unconstrained statuses might be correlated with export decisions. Finally, we run a placebo test to check the accuracy of the identification of the firms classified as financially constrained.

#### Propensity score matching analysis

To eliminate any suspicions that the results might be driven by systematic differences between financially constrained and unconstrained firms, we perform a propensity score matching analysis. We match each financially constrained firm with an unconstrained one without replacement and with a calliper of 0.5%. The analysis takes into consideration financially constrained firms as the treatment group, and the unconstrained ones as the control group, since the latter contains more observations. The matching approach selects matches minimizing the difference between the propensity scores between the treated and the control group. We perform a logistic regression of each financial constraint dummy variable, matching on all covariates, including age, age-squared, cash flow to capital, leverage, productivity, GDP growth and Covid.

**Table 5:** Propensity score matching analysis

	$\textrm{SAD}_{it}$		$\textrm{WWD}_{it}$		$\textrm{IBD}_{it}$	
	ATE	ATET	ATE	ATET	ATE	ATET
	(1)	(2)	(3)	(4)	(5)	(6)
$$\textrm{Ln}(E_{it})$$	$$-$$ 0.535$$^{***}$$	$$-$$ 0.548$$^{***}$$	$$-$$ 0.501$$^{***}$$	$$-$$ 0.506$$^{***}$$	$$-$$ 0.514$$^{***}$$	$$-$$ 0.481$$^{***}$$
	(0.058)	(0.060)	(0.058)	(0.063)	(0.057)	(0.053)
$X^{'}_{i,t-1}$	Yes	Yes	Yes	Yes	Yes	Yes
Industry FE	Yes	Yes	Yes	Yes	Yes	Yes
Year FE	Yes	Yes	Yes	Yes	Yes	Yes
N	109,794	109,794	109,794	109,794	109,794	109,794

This table reports the results of the propensity score matching on firms’ exports. The estimates are conducted by matching each financially constrained firm to an unconstrained firm using one-to-one propensity score matching to the nearest neighbourhood, without replacement. The average treatment effects (ATE) estimates the average effects of financial constraints in the population. The treatment effects on the treated (ATET) estimates the impact of financial constraints on constrained firms, i.e. the treated group. The dependent variable is the natural logarithm of exports ($$\textrm{Ln}(E_{it})$$). The matching is based on all the control variables, including $$\textrm{Age}_{i,t-1}$$, $$(\textrm{Age}_{i,t-1})^{2}$$, $$\textrm{Ln}(\textrm{Emp}_{i,t-1})$$, $$\textrm{CFK}_{i,t-1}$$, $$\textrm{LEV}_{i,t-1}$$, $$\textrm{Ln}(\textrm{TFP}_{i,t-1})$$, $$\textrm{Covid}_{t}$$, $$\textrm{GDP}_{h,t-1}$$, which are defined in Table 1. This table displays three measures of financial constraints ($$F_{i,t-1}$$): (i) $$\textrm{SAD}_{i,t-1}$$ is a dummy variable which equals one for observations with the Hadlock and Pierce (2010) SA index above the 1/3 percentile, and zero otherwise; (ii) $$\textrm{WWD}_{i,t-1}$$ is a dummy variable which equals one for observations with the Whited and Wu (2006) index above the 1/3 percentile, and zero otherwise; and the $$\textrm{IBD}_{i,t-1}$$ indicator is a dummy variable which equals one for observation with the interest-paid-to-cash-flow ratio ($$\textrm{IB}_{i,t-1}$$) above the 1/3 percentile, and zero otherwise. Bootstrapped standard errors are in parentheses. Estimates followed by $$^{*}$$, $$^{**}$$, $$^{***}$$ are statistically significant at the 5%, 1% and 0.1% levels, respectively

Table 5 presents the regression results for the propensity-scored samples. The negative and significant signs suggest that financially constrained firms are unable to export as unconstrained firms do. Interestingly, the magnitude of the coefficients on the three financial constraint dichotomous indicators $\left( F_{it}\right)$ are even larger than in the baseline regression, indicating that the impact of being credit-constrained is economically significant.

#### Transition sample analysis

This section performs a complementary endogeneity test to alleviate concerns arising from firm heterogeneity such as the case of ‘transitioning’ firms that switch from unconstrained to constrained status over the sample period. This approach allows us to control for time-invariant unobservable industry characteristics and time-trend effects which may be correlated with export decisions. First, we focus on financially constrained firms and perform regression analysis to investigate variations in exports following the financial constraint event. Subsequently, we include temporary and permanent effects of firms’ financial constraint events on exports in the equation for exports’ amounts of the Heckman (1979) models as follows:

4 $$\begin{aligned} ExpD_{it}= & {} \left\{ \begin{array}{ll} 1 &{}\quad \text {if } \gamma ^{a}_{0}ExpD_{i,t-1} + \gamma ^{a}_{1}F^{I}_{i,t} + Z_{i,t-1}^{'}\Gamma ^{a} + \nu _{k} + \tau _{t} + \omega _{it} \ge 0 \\ 0 &{}\quad \text {otherwise} \end{array}\right. \end{aligned}$$

5 $$\begin{aligned} \textrm{Ln}(E_{i,t})= & {} \phi _{0} + \phi _{1} F^{I}_{i,t} + X_{i,t-1}^\prime \Phi +\nu _{k}+\tau _{t}+\epsilon _{i,t} \end{aligned}$$

6 $$\begin{aligned} ExpD_{it}= & {} \left\{ \begin{array}{ll} 1 &{}\quad \text {if } \gamma ^{b}_{0}ExpD_{i,t-1} + \gamma ^{b}_{1}F^{I}_{T} + \sum _{j=1}^{3} \gamma ^{b}_{j+1}F^{I}_{i,T+j} + \gamma ^{b}_{5}F^{I}_{T \ge 4} \\ &{}\quad + Z_{i,t-1}^{'}\Gamma ^{b} + \nu _{k} + \tau _{t} + \omega _{it} \ge 0 \\ 0 &{}\quad \text {otherwise} \end{array}\right. \end{aligned}$$

7 $$\begin{aligned} \textrm{Ln}(E_{i,t})= & {} \phi ^{'}_{0} + \phi ^{'}_{1}F^{I}_{T} + \sum _{j=1}^{3} \phi ^{'}_{j+1}F^{I}_{i,T+j} + \phi ^{'}_{5}F^{I}_{T \ge 4} + X_{i,t-1}^\prime \Phi +\nu _{k}+\tau _{t}+\epsilon _{i,t}\nonumber \\ \end{aligned}$$

Let T be the year when the firm becomes constrained for the first time, so $F_{i,t}^{I}$ is a dummy variable that takes value one for any year $t\ge T$. In models (6) and (7), $F_{i,T+1}^{I }$ (respectively $F_{i,T+2}^{I }$ and $F_{i,T+2}^{I }$ ) is a dummy variable that takes value one, one year (respectively two and three years) after firm *i* became financially constrained and zero otherwise. Lastly, $F_{i,T \ge 4}^{I}$ is a dummy variable that takes value one during the four years after the firm became financially constrained, and zero otherwise.

Both models include the usual set of control variables ($Z_{i,t-1}$ and $X^{'}_{i,t-1}$), industry ($\nu _{k}$) and year ($\tau _{t}$) fixed effects. In these models, financially constrained firms are considered part of the treatment group, whereas the unconstrained ones are part of the control group. Consistently, the coefficients $\gamma ^{a}_{1}$, $\phi _{1}$, $\gamma ^{b}_{1}$, $\phi ^{'}_{1}$, $\gamma ^{b}_{2}$, $\gamma ^{b}_{3}$, $\gamma ^{b}_{4}$, $\gamma ^{b}_{5}$, $\phi ^{'}_{2}$, $\phi ^{'}_{3}$, $\phi ^{'}_{4}$ and $\phi ^{'}_{5}$, are expected to be negative and significant.

**Table 6:** Transition sample analysis

	$\textrm{SAD}_{it}$	$\textrm{WWD}_{it}$	$\textrm{IBD}_{it}$	$\textrm{SAD}_{it}$	$\textrm{WWD}_{it}$	$\textrm{IBD}_{it}$
	(1)	(2)	(3)	(4)	(5)	(6)
*Panel A: Export decision (average marginal effects)*						
$$ExpD_{i,t-1}$$	0.322$$^{***}$$	0.315$$^{***}$$	0.321$$^{***}$$	0.322$$^{***}$$	0.315$$^{***}$$	0.321$$^{***}$$
	(0.002)	(0.002)	(0.001)	(0.002)	(0.002)	(0.001)
$$\textrm{CCC}_{i,t-1}$$	$$-$$ 0.001$$^{***}$$	$$-$$ 0.001$$^{***}$$	$$-$$ 0.001$$^{***}$$	$$-$$ 0.001$$^{***}$$	$$-$$ 0.001$$^{***}$$	$$-$$ 0.001$$^{***}$$
	(0.000)	(0.000)	(0.000)	(0.000)	(0.000)	(0.000)
$$F^{I}_{i,t}$$	$$-$$ 0.012$$^{***}$$	$$-$$ 0.018$$^{***}$$	$$-$$ 0.013$$^{***}$$			
	(0.001)	(0.002)	(0.001)			
$$F^{I}_{i,T+1}$$				$$-$$ 0.012$$^{***}$$	$$-$$ 0.018$$^{***}$$	$$-$$ 0.009$$^{***}$$
				(0.001)	(0.002)	(0.002)
$$F^{I}_{i,T+2}$$				$$-$$ 0.011$$^{**}$$	$$-$$ 0.014$$^{***}$$	$$-$$ 0.007$$^{***}$$
				(0.001)	(0.001)	(0.001)
$$F^{I}_{i,T+3}$$				$$-$$ 0.010$$^{**}$$	$$-$$ 0.010$$^{**}$$	$$-$$ 0.006$$^{***}$$
				(0.003)	(0.003)	(0.002)
$F^{I}_{i,T\ge 4}$				$-$ 0.002	$-$ 0.002	$-$ 0.000
				(0.006)	(0.002)	(0.002)
$Z_{i,t-1}^{'}$	Yes	Yes	Yes	Yes	Yes	Yes
Industry FE	Yes	Yes	Yes	Yes	Yes	Yes
Year FE	Yes	Yes	Yes	Yes	Yes	Yes
*Panel B: Export volume*						
$$F^{I}_{i,t}$$	$$-$$ 0.164$$^{***}$$	$$-$$ 0.136$$^{***}$$	$$-$$ 0.088$$^{***}$$			
	(0.020)	(0.014)	(0.012)			
$$F^{I}_{i,T}$$				$$-$$ 0.159$$^{***}$$	$$-$$ 0.154$$^{***}$$	$$-$$ 0.089$$^{***}$$
				(0.024)	(0.018)	(0.015)
$$F^{I}_{i,T+1}$$				$$-$$ 0.124$$^{***}$$	$$-$$ 0.134$$^{***}$$	$$-$$ 0.056$$^{***}$$
				(0.013)	(0.017)	(0.018)
$$F^{I}_{i,T+2}$$				$$-$$ 0.118$$^{***}$$	$$-$$ 0.129$$^{***}$$	$$-$$ 0.053$$^{***}$$
				(0.039)	(0.014)	(0.020)
$$F^{I}_{i,T+3}$$				$$-$$ 0.103$$^{**}$$	$$-$$ 0.104$$^{**}$$	$$-$$ 0.039$$^{**}$$
				(0.041)	(0.040)	(0.019)
$F^{I}_{i,T\ge 4}$				$-$ 0.057	$-$ 0.019	0.016
				(0.041)	(0.021)	(0.019)
$$\lambda _{it}$$	0.921$$^{***}$$	0.921$$^{***}$$	0.921$$^{***}$$	0.921$$^{***}$$	0.921$$^{***}$$	0.921$$^{***}$$
	(0.009)	(0.009)	(0.009)	(0.009)	(0.009)	(0.009)
$X^{'}_{i,t-1}$	Yes	Yes	Yes	Yes	Yes	Yes
Industry FE	Yes	Yes	Yes	Yes	Yes	Yes
Year FE	Yes	Yes	Yes	Yes	Yes	Yes
N	109,794	109,794	109,794	109,794	109,794	109,794
Wald’s test [*p* values]	0.000	0.000	0.000	0.000	0.000	0.000
LR test [*p* values]	0.000	0.000	0.000	0.000	0.000	0.000

This table reports the results for the transition sample of 13,727 firms. Annual observations for British, Croatian, Estonian, French, German, Hungarian, Irish and US firms are applied from 2012 to 2020. The dependent variable is the natural logarithm of exports ($$\textrm{Ln}(E_{it})$$). This table displays three measures of financial constraints: (i) $$\textrm{SAD}_{i,t-1}$$ is a dummy variable which equals one for observations with the Hadlock and Pierce (2010) SA index above the 1/3 percentile, and zero otherwise; (ii) $$\textrm{WWD}_{i,t-1}$$ is a dummy variable which equals one for observations with the Whited and Wu (2006) index above the 1/3 percentile, and zero otherwise; and the $$\textrm{IBD}_{i,t-1}$$ indicator is a dummy variable which equals one for observation with the interest-paid-to-cash-flow ratio ($$\textrm{IB}_{i,t-1}$$) above the 1/3 percentile, and zero otherwise. The variable $$F_{i,t}^{I}$$ is a dummy variable that takes the value one since the firm becomes financially constrained thenceforth. $$F_{i,T+j}^{I}$$ with $$j={1,2,3}$$ is a dummy variable that takes the value one in the year when the firm is financially constrained, and zero otherwise. $$F_{i,T \ge 4}^{I}$$ is a dummy that takes the value one for the period at least four years after the financial constraint event. The matrix $$Z_{i,t-1}^{'}$$ includes the following control variables: $$\textrm{Ln}(\textrm{Age}_{i,t-1})$$, $$\textrm{Ln}(\textrm{Age}_{i,t-1})^{2}$$, $$\textrm{Ln}(\textrm{Emp}_{i,t-1})$$, $$\textrm{Ln}(\textrm{TFP}_{i,t-1})$$, $$\textrm{Covid}_{t}$$. The matrix $$X_{i,t-1}^{'}$$ adds to $$Z_{i,t-1}^{'}$$ the following variables: $$\textrm{CFK}_{i,t-1}$$, $$\textrm{LEV}_{i,t-1}$$, $$\textrm{GDP}_{h,t-1}$$. All the variables are defined in Table 1. The estimations are conducted using the Heckman (1979) estimator. Goodness of fit is tested using the Wald test (*p* values). Independence between the regression and the selection equations is tested using the LR test (*p* values) under the null of no selection process ($$H_{0}: \rho =0$$). Robust standard errors are presented in parentheses (White 1980). Estimates followed by $$^{*}$$, $$^{**}$$, $$^{***}$$ are statistically significant at the 5, 1 and 0.1% levels, respectively

Table 6 presents the regression results for the firms that became financially constrained during the sample period. Columns (1)–(3) report the results for Eqs. (4) and (5). As expected, estimates on $F_{i,t}^{I}$ (i.e. coefficients $\phi _{1}$ and $\gamma ^{a}_{1}$) are negative and significant for the three financial constraint indicators—both in the selection equation and the equation for volume—which is consistent with previous research demonstrating that firms becoming constrained export comparatively less than unconstrained firms (e.g. Minetti and Zhu 2011; Minetti et al. 2018; Muûls 2015; Pietrovito and Pozzolo 2021). In addition, columns (4)–(6) display regression results for models (6) and (7). The negative and significant estimates on $F^{I}_{i,T+j}$ (i.e. coefficients $\phi ^{'}_{j+1}$ and $\gamma ^{b}_{j+1}$) confirm previous conjectures that financial constraints might have a persistent impact over time both on the extensive and intensive margins. Nevertheless, the magnitude of the coefficients diminishes over time, indicating that the impact of financial constraints on firms’ exports is concentrated in the first years following the event.

#### Placebo regression

It might be argued that the above-discussed results are driven by misidentification issues of financially constrained firms due to inaccuracy in the classification criteria. To alleviate concerns about imprecise estimations, we estimate a placebo regression for the whole experiment conducted in this research. For this purpose, we create the dummy variable $P_{i,t-1}$ that divides randomly financially constrained and unconstrained firms across the sample. The estimates on $P_{i,t-1}$ are expected to be insignificant. Otherwise, previous results would have been as near random as those presented in this section.

**Table 7:** Placebo regression

	$\textrm{CCC}_{i,t-1}$		$\textrm{TC}_{i,t-1}$		$\textrm{INV}_{i,t-1}$		$\textrm{TD}_{i,t-1}$	
	(1)	(2)	(3)	(4)	(5)	(6)	(7)	(8)
*Panel A: Export decision (average marginal effects)*								
$$ExpD_{i,t-1}$$	0.322$$^{***}$$	0.322$$^{***}$$	0.322$$^{***}$$	0.322$$^{***}$$	0.324$$^{***}$$	0.324$$^{***}$$	0.325$$^{***}$$	0.325$$^{***}$$
	(0.002)	(0.002)	(0.002)	(0.002)	(0.002)	(0.002)	(0.002)	(0.002)
$$I_{i,t-1}$$	$$-$$ 0.001$$^{***}$$	$$-$$ 0.001$$^{***}$$	$$-$$ 0.001$$^{***}$$	$$-$$ 0.001$$^{***}$$	$$-$$ 0.001$$^{***}$$	$$-$$ 0.001$$^{***}$$	0.001$$^{***}$$	0.001$$^{***}$$
	(0.000)	(0.000)	(0.000)	(0.000)	(0.000)	(0.000)	(0.000)	(0.000)
$P_{i,t-1}$	$-$ 0.000	0.000	0.000	0.000	0.000		0.000	0.000
	(0.000)	(0.000)	(0.000)	(0.000)	(0.000)		(0.000)	(0.000)
$I_{i,t-1} \times P_{i,t-1}$		0.000		0.000		0.000		0.000
		(0.000)		(0.000)		(0.000)		(0.000)
$Z_{i,t-1}^{'}$	Yes	Yes	Yes	Yes	Yes	Yes	Yes	Yes
Industry FE	Yes	Yes	Yes	Yes	Yes	Yes	Yes	Yes
Year FE	Yes	Yes	Yes	Yes	Yes	Yes	Yes	Yes
*Panel B: Export volume*								
$$I_{i,t-1}$$	$$-$$ 0.001$$^{***}$$	$$-$$ 0.001$$^{***}$$	$$-$$ 0.001$$^{***}$$	$$-$$ 0.001$$^{***}$$	$$-$$ 0.001$$^{***}$$	$$-$$ 0.001$$^{***}$$	0.001$$^{***}$$	0.001$$^{***}$$
	(0.000)	(0.000)	(0.000)	(0.000)	(0.000)	(0.000)	(0.000)	(0.000)
$P_{i,t-1}$	$-$ 0.000	0.000	0.000	0.000	0.000		0.000	0.000
	(0.000)	(0.000)	(0.000)	(0.000)	(0.000)		(0.000)	(0.000)
$I_{i,t-1} \times P_{i,t-1}$		0.000		0.000		0.000		0.000
		(0.000)		(0.000)		(0.000)		(0.000)
$$\lambda _{it}$$	0.913$$^{***}$$	0.913$$^{***}$$	0.913$$^{***}$$	0.913$$^{***}$$	0.913$$^{***}$$	0.913$$^{***}$$	0.913$$^{***}$$	0.913$$^{***}$$
	(0.009)	(0.009)	(0.009)	(0.009)	(0.009)	(0.009)	(0.009)	(0.009)
$X_{i,t-1}^{'}$	Yes	Yes	Yes	Yes	Yes	Yes	Yes	Yes
Industry FE	Yes	Yes	Yes	Yes	Yes	Yes	Yes	Yes
Year FE	Yes	Yes	Yes	Yes	Yes	Yes	Yes	Yes
*N*	109,794	109,794	109,794	109,794	109,794	109,794	109,794	109,794
Wald’s test [*p* values]	0.000	0.000	0.000	0.000	0.000	0.000	0.000	0.000
LR test [*p* values]	0.000	0.000	0.000	0.000	0.000	0.000	0.000	0.000

This table reports the placebo tests on firms’ exports. Annual observations for British, Croatian, Estonian, French, German, Hungarian, Irish and US firms are applied from 2012 to 2020. The dependent variable is the natural logarithm of exports ($$\textrm{Ln}(E_{it})$$). $$I_{i,t-1}$$ refers to the cash conversion cycle ($$\textrm{CCC}_{i,t-1}$$) (Columns (1)–(2)), the credit period ($$\textrm{TC}_{i,t-1}$$) (Columns (3)–(4)), the inventory period ($$\textrm{INV}_{i,t-1}$$) (Columns (5)–(6)) and the collection period ($$\textrm{TD}_{i,t-1}$$) (Columns (7)–(8)). We randomly classify firms as financially constrained and unconstrained firms using the placebo indicator $$P_{i,t-1}$$. The matrix $$Z_{i,t-1}^{'}$$ includes the following control variables: $$\textrm{Ln}(\textrm{Age}_{i,t-1})$$, $$\textrm{Ln}(\textrm{Age}_{i,t-1})^{2}$$, $$\textrm{Ln}(\textrm{Emp}_{i,t-1})$$, $$\textrm{Ln}(\textrm{TFP}_{i,t-1})$$, $$\textrm{Covid}_{t}$$. The matrix $$X_{i,t-1}^{'}$$ adds to $$Z_{i,t-1}^{'}$$ the following variables: $$\textrm{CFK}_{i,t-1}$$, $$\textrm{LEV}_{i,t-1}$$, $$\textrm{GDP}_{h,t-1}$$. All the variables are defined in Table 1. The estimations are conducted using the Heckman (1979) estimator. Goodness of fit is tested using the Wald test (*p* values). Independence between the regression and the selection equations is tested using the LR test (*p* values) under the null of no selection process ($$H_{0}: \rho =0$$). Robust standard errors are presented in parentheses (White 1980). Estimates followed by $$^{*}$$, $$^{**}$$, $$^{***}$$ are statistically significant at the 5%, 1% and 0.1% levels, respectively

Table 7 displays the results for the placebo regression. As expected, the estimated estimates on $P_{i,t-1}$ are not significant in any of the estimations, suggesting that there is no coincidence between the former variable and the financial constraint indicators $\left( F^{c}_{it}\right)$. Consequently, we can assume that the coefficient estimates of the baseline regression model are valid.

## Conclusions

This article draws attention to the role of working capital management in explaining the export behaviour of firms, a question that has been largely overlooked by the literature even if working capital management has proven to be crucial to maintain firms’ financial equilibrium, in particular when financial markets are imperfect. The key results of this article suggest that if firms manage their working capital inadequately, which translates to a longer cash conversion cycle (CCC), they would reduce their chances of exporting and the volume of their exports. The results are robust to sample selection, endogeneity and placebo tests. These conclusions also apply when we consider each component of the CCC. Indeed, delays in collecting receivables and selling stocks create an opportunity cost that might impede firms from expanding their capacity to produce and expand their horizons because they might postpone investments in fixed capital that could jeopardize their capacity for production and export (see Biais and Gollier 2015; Petersen and Rajan 1996). Nonetheless, lengthening payable periods, which is an inexpensive source of finance (Long et al. 1993) and a signal of creditworthiness (Doan et al. 2020), contributes to fostering exports.

The second pillar of this paper built on previous research that demonstrates that the lack of external financing—namely, financial constraints—prevents firms from exporting. An additional contribution of our study is to confirm these results for a large sample of US and European firms, while most previous studies tackle this issue for samples of developing and emerging countries or focus only on one specific developed country.

We go one step further by raising the question of whether the CCC-exports relationship is comparatively sharper for financially constrained firms than for unconstrained firms. We find that the shortage of working capital is an even more binding factor for exports for financially constrained compared with unconstrained firms. In other words, the lack of external financing might exacerbate firms’ liquidity shortages. The explanation of the transmission channel under investigation proceeds as follows. Shortages of liquidity resources, which prevent firms from exporting, need to be solved by applying to external finance, e.g. line of credits, export letters of credits, among others (Antràs and Foley 2015; Niepmann and Schmidt-Eisenlohr 2017). If a firm with such troubles cannot borrow the desired amount of credit to solve them, it will be unable to produce enough to approach foreign markets.

In short, internationalization requires simultaneously internal and external financial resources. Firms failing to meet one of these requirements become economically more vulnerable to external shocks. This is a very timely question in the aftermath of the COVID-19 crisis which caused an unprecedented decline in international trade and a rise in firms’ need for liquidity. The results of this study should prove interesting for scholars, entrepreneurs and policymakers for four reasons. First, the breakthrough of this research is that high-CCC firms are less likely to expand their markets beyond their domestic frontiers. Second, balancing working capital could be a powerful tool for internationalizing firms, at least at the level of exports. Third, in the light of our results, regulators and policymakers should be aware that the flow of the payment chain might be a further tool for fostering exports, which are a powerful engine of economic growth. Lastly, responding promptly to financial crises so as to avoid scarcity of credit to the real sector might attenuate the impact of shortage of liquidity on exports.

## Appendix A

See Table 8.

**Table 8:** NACE manufacturing sectors

Acronym	Sector
C10	Food products
C11	Beverages
C12	Tobacco products
C13	Textiles
C14	Wearing apparel
C15	Leather and related products
C16	Wood and of products of wood and cork, except furniture; articles of straw and plaiting materials
C17	Paper and paper products
C18	Printing and reproduction of recorded media
C19	Coke and refined petroleum products
C20	Chemicals and chemical products
C21	Basic pharmaceutical products and pharmaceutical preparations
C22	Rubber and plastic products
C23	Other non-metallic mineral products
C24	Basic metals
C25	Fabricated metal products, except machinery and equipment
C26	Computer, electronic and optical products
C27	Electrical equipment
C28	Machinery and equipment n.e.c
C29	Motor vehicles, trailers and semi-trailers
C30	Other transport equipment
C31	Furniture
C32	Other manufacturing

## Footnotes

1 See Altavilla et al. (2020) for an in-depth discussion on the ECB’s response policies targeted at improving bank lending conditions to mitigate the impact of the COVID-19 crisis.

2 Some exceptions are Greenaway et al. (2007), Bricongne et al. (2012), Muûls (2015), Minetti and Zhu (2011) and Minetti et al. (2018).

3 Foley and Manova (2015) on page 15 explains that “under cash in-advance terms, importers fund the working capital needs by paying exporters before goods are shipped. Under open-account terms, exporters fund the working capital needs and allow importers to pay at a pre-specified time after the goods have arrived at their destination”.

4 Access to letters of credit helps firms to alleviate information asymmetries in foreign markets, in particular in countries with relatively poor financial or institutional development. In times of crisis, the effect of letter-of-credit supply shocks has particularly greater repercussions on exporters than other forms of credit due to (i) it being trade-specific and (ii) there being fewer banks willing to change market destinations, so creating switching costs for exporters (Antràs and Foley 2015; Niepmann and Schmidt-Eisenlohr 2017).

5 Studies dealing with trade finance show that trade credit might be comparatively more expensive if the firm is unable to externally finance its working capital, i.e. if the firm is financially constrained (e.g. Biais and Gollier 2015; Petersen and Rajan 1996). Interestingly, trade creditors are found to deal with information asymmetry and mitigate weak creditor protection comparatively better than banks (Ivashina and Iverson 2018), which bolsters transactions in countries with a relatively underdeveloped financial system (e.g. Burkart and Ellingsen 2004; Fisman and Love 2003).

6 In this regard, financial literature is hotly debating the role of cash-flow sensitivity on financial constraints. The seminal papers presented by Fazzari et al. (1988) and Fazzari and Petersen (1993) show that financial constraints are closely related to cash flow sensitivities. This argument is in line with Kaplan and Zingales (1997, 2000), who suggest that cash-flow sensitivity should not necessarily be interpreted as a sign of financial constraints. Similarly, Cleary (1999, 2006) finds that cash-flow sensitivity need not identify liquidity-constrained firms. Importantly, there are authors who demonstrate that maintaining certain levels of liquidity might be determinant, particularly when financial markets are imperfect (Blanchard et al. 1994; Kim et al. 1998; Lins et al. 2010; Yun 2009).

7 The Orbis database is built from information from several national sources covering a broad range of balance sheets and managerial information, which makes it suitable for research in corporate finance. The main advantage of Orbis is that accountant information is homogenized across countries, and unique identification codes are assigned to each firm from its entry into the database. Consequently, each firm can be tracked over time, enabling researchers to exploit panel data econometric techniques. Nevertheless, although Orbis is biased towards large companies, the representativeness of the sample is not affected since it covers a large set of manufacturers in the OECD countries (Fariñas et al. 2018; Weche 2018).

8 Interestingly, let us highlight the contribution of the aforementioned activity ratios to the sign of the cash conversion cycle ($\textrm{CCC}_{it}$). First, accumulating accounts receivable, which are essentially loans to customers, might make the company lose investment opportunities as long as the money is unavailable. Secondly, if managers fail to realize potential sales, thus accumulating inventories, cash is tied up in goods that cannot be sold, which might make them decide to reduce the price of such products at a loss. Lastly, the firm benefits from slowing down payments of accounts payable to its suppliers because such ‘extra time’ allows it to make use of money longer.

9 This criterion has been extensively used in the financial literature (see Collier et al. 2020; Linck et al. 2013; Smith 2014).

10 This criterion has been extensively used in the financial literature (see Linck et al. 2013; Smith 2014).

11 There is a broad branch of the literature demonstrating that the interest burden ratio exerts a relevant impact on firms’ real decisions. In this regard, previous research reveals that firms putting up with relatively higher levels of IB might reduce their number of employees (Spaliara 2009; Chen and Guariglia 2013; Fernandes et al. 2019), and their likelihood of survival (Guariglia et al. 2016).

12 Heckman’s selection model assumes that the error terms of Eqs. (2) and (3) are correlated ($Corr(\omega _{it}, u_{it}) = \rho$). If $\rho \ne 0$, the standard regression techniques applied to Eq. (3) yield biased results. Thus, we test the orthogonality between the selection and the regression equations using the likelihood ratio (LR hereafter) test under the null of no selection process ($H_{0}: \rho = 0$). Rejecting the null implies that Heckman’s estimator provides consistent, asymptotically efficient estimates for the whole set of parameters in such models.

13 The Wald test rejects the null for all the specifications, thus confirming the significance of the overall model. Importantly, the LR test also rejects the null of no selection process ($H_{0}: \rho = 0$), thus confirming the adequacy of Heckman’s estimator.
